# Supplementary material for: The earliest known titanosauriform sauropod dinosaur and the evolution of Brachiosauridae
Source: PeerJ. 2017 May 2;5:e3217. doi: 10.7717/peerj.3217 (PMC5417094; doi:10.7717/peerj.3217)
Supplement: Supplemental Information 1 [file peerj-05-3217-s001.doc]

**Supplementary Information**

**STRATIGRAPHIC POSITION**

UNCERTAINTY CONCERNING THE LAST ESTABLISHED AGE

The exact correlation of the *calcaires fins* with the two members of the *Formation de Clerval* remains unclear in the regional synthesis of Enay, Contini & Boullier (1988). In lithological descriptions, the *Calcaires de l’Isle-sur-le-Doubs* Member is described as “very reduced around Dole”, with the Damparis fossil assemblage at its base, and the *Tidalites de Mouchard* Member “does not quite reach 10 m in thickness at Damparis, near Dole”. In Enay, Contini & Boullier (1988: fig. 5), these two members are distinguished and directly superimposed at the top of the *Oolite coralienne* Formation in the summarized Dole profile. In contrast, eastward from Dole, these two members are merged into a single unit incorrectly named “*Calc. de l’Isle*” in Contini (1989: fig. 6), but correctly named *Calcaires de Clerval* in Contini (1989: fig. 2). Consequently, both *Calcaires de l’Isle-sur-le-Doubs* and *Tidalites de Mouchard* members seem to have been correlated with the *calcaires fins*, but without specifying the exact position of their limit within this local formation (Enay, Contini & Boullier, 1988).

At Damparis, the discovery of an ammonite *Perisphinctes* (*Ampthillia*) cf. *quadratus* in the overlying *calcaires graveleux inférieur* led to the placement of this unit in the *Bifurcatus* zone (Boullier, Contini & Pernin, 1975), as well as the base of the *Marnes de Besançon* Formation, to which they were correlated by Enay, Contini & Boullier (1988). However, the absence of ammonites in the *calcaires fins* at Damparis and in the *Tidalites de Mouchard* at the regional scale maintains some doubt as to whether both units belong to the top of the *Transversarium* zone or to the base of the *Bifurcatus* zone. Enay, Contini & Boullier (1988) used the significant increase in terrigenous inputs observed on the platform and in the basin to: (1) correlate the base of the *Marnes de Besançon* and the base of the *Couches d’Effingen* (p. 341), and (2) define the limit between major lithoclinal sequences S1 and S2 (p. 347). The *Calcaires de Clerval* Formation then positioned at the end of S1 was thus correlated with the top of the *Couches de Birmenstorf*, with common evidence of limited terrigenous inputs (Enay, Contini & Boullier, 1988: p. 327). The dating of the S1–S2 limit at the transition between the *Transversarium* and *Bifurcatus* zones led to the attribution of the *Calcaires de l’Isle-sur-le-Doubs*, and thus the Damparis fossils, to the top of the *Transversarium* zone (Enay, Contini & Boullier: p. 302; Contini, 1989). This last opinion – positioning the Damparis fossils in the late middle Oxfordian – was followed by Broin *et al*. (1992) and Buffetaut (1994). However, as the *Calcaires de l’Isle-sur-le-Doubs* has not been clearly identified at Damparis, and may even be absent, the attribution of the fossils to the end of the S1 lithoclinal sequence and thus to the top of the *Transversarium* zone remains uncertain.

UPDATED CORRELATIONS

A few fragments of ammonites recently found in the *calcaires hydrauliques* at the base of the Damparis quarry (Tables S1, S2) strengthen its biochronological context, but do not refine the age of the fossil assemblage. The use of cyclostratigraphy and the revision of the correlations of the Damparis geological profile lead us to propose a slightly different chronological attribution of the Damparis fossils.

**Cyclostratigraphy**

Recent sequence stratigraphy analyses performed on the Swiss platform (Gygi, Coe & Vail, 1998; Strasser, 2007; Comment & Ayer, 2010) identified up to six third order cycle sequence boundaries (Ox #) throughout the Middle–Upper Oxfordian sequence. Ox 5 is positioned at the top of the Sainte-Ursanne Formation. On the platform, Ox 6 is positioned in the upper part of the Röschenz Member, at the base of marine marls transgressing over peritidal deposits and ending with an ooid marker bed correlated with the Grüne Mumienbank bed in the basin (Gygi & Persoz, 1986; Gygi, Coe & Vail, 1998). Ox 6 is often associated with evidence for emersion of the platform and a renewed input of argillaceous marls in the basin (Gygi, Coe & Vail, 1998). Additionally, Ox 6 is synchronous with a second order cycle sequence boundary (Hardenbol *et al*., 1998). Together with Ox 5, they bracket a third order cycle encompassing the Vorbourg Member, whose upper part is equivalent to the *Tidalites de Mouchard* (Enay, Contini & Boullier, 1988).

Identified at the top of the *Oolithe corallienne de Pagnoz* (Strasser, 2007), Ox 5 occurs at the same stratigraphic position as in in Switzerland. At Damparis, the Ox 5 conspicuously takes place at the limit between the *calcaires massifs crayeux bioclastiques* and the *calcaires fins* (Table S1; Fig. 6). The presence of *Perisphinctes* (*Dichotomoceras*) *wartae* and *Larcheria* sp. in the *calcaires hydrauliques* at Damparis, and in other local quarries (Tables S1, S2), implies that Ox 5 occurred before the top of the *Schilli* subzone, in agreement with the position of the *Calcaires de l’Isle-sur-le-Doubs* at the end of the *Transversarium* zone (Fig. 6). In several locations, Ox 5 is associated with a truncation at the base of a reduced *Calcaires de Clerval* Formation (Enay, Contini & Boullier, 1988; Fig. S1), and is thus synchronous with the first terrigenous inputs on the platform. Consequently, quartz grains found at the top of the *Couches de Birmenstorf* in the French Jura basin are not necessarily contemporaneous with the *Calcaires de l’Isle-sur-le-Doubs* Member (Enay, Contini & Boullier, 1988), but could result from early local erosions of surrounding emerged lands, possibly after the *Schilli* subzone. Such a chronology is in agreement with the presence of *Larcheria schilli* at the base of the Effingen Beds in Switzerland (Gygi, 2012: fig. 1.5). Besides, the lithological context of the Ox 6 sequence boundary on the platform in Switzerland (see above) led to its association with the bored surface at Damparis that defines the boundary between the peritidal *calcaire graveleux inférieurs* and the marine *marnes et calcaires*, accompanied by a return of the benthic fauna (Boullier, Contini & Pernin, 1975) (Tables S1; Fig. 7). This position of Ox 6, dated from the *Bifurcatus*–*Bimammatum* limit (Gygi, Coe & Vail, 1998), is consistent with the discovery of the ammonite *Perisphinctes* cf. *quadratus* at the base of the *graveleux inférieurs*.

**Cyclostratigraphy vs. sedimentology**

Focusing now between Ox 5 and Ox 6 in the *calcaires fins*, the main observation is the 10% concentration in quartz grains recorded in the sediment embedding the Damparis fossils (Dreyfuss, 1934) and in the basal clay bed of the *calcaires fins*. Originally used to define the basis of the outdated “Sequanian”, the arrival of terrigenous inputs (quartz grains and clays) on the platform is used to distinguish the *Tidalites de Mouchard* from the *Calcaires de l’Isle-sur-le-Doubs*, which lacks these elements (Enay, Contini & Boullier, 1988). A detailed study of the stratigraphic distribution of quartz inputs within the *Calcaires de Clerval* Formation would however be useful to confirm the reliability of this discriminant marker at the regional scale. Boullier, Contini & Pernin (1975) and Pernin (1978) only mentioned the clayey beds in their description of the *calcaires fins* at Damparis. Considered “very reduced around Dole” (Enay, Contini & Boullier, 1988), the *Calcaires de l’Isle-sur-le-Doubs* could actually be absent from the *calcaires fins* at Damparis. Consequently, contrary to what has been indicated by Enay, Contini & Boullier (1988), the bones and teeth of the Damparis dinosaurs would have thus been found in the *Tidalites de Mouchard* Member. Such a hypothesis is supported by evidence of recurrent emersion phases and a very poor/ammonite-free marine fauna in this member at both local (Boullier, Contini & Pernin, 1975) and regional (Enay, Contini & Boullier, 1988; Contini, 1989) scales, as well as in Switzerland (Gygi, 2012). So, as far as the *Calcaires de l’Isle-sur-le-Doubs* was well distinguished from the uppermost units of the *Oolithe coralienne de Pagnoz*, especially in the case of old samplings, stratigraphic ranges of ammonites indicate that the deposition of the *Calcaires de l’Isle-sur-le-Doubs* in the region occurred at the top of the *Transversarium* zone (Enay, Contini & Boullier, 1988), i.e. in the *Rotoides* subzone (Table S2).

In the subsequent *calcaires graveleux inférieurs*, quartz grain concentrations are definitely higher (Boullier, Contini & Pernin, 1975; Pernin, 1978), supporting correlations with the basal part of the *Marnes de Besançon* (Enay, Contini & Boullier, 1988), but also with the Röschenz Member (20–50%; in contrast to the 10% in the underlying Vorbourg Member) in Switzerland (Bolliger & Burri, 1967; Gygi, 2000).

**Biostratigraphy**

At Damparis, the ammonite *Perisphinctes* cf. *quadratus*, typical of the *Bifurcatus* zone, has been found at the base of the *calcaires graveleux inférieurs* (Boullier, Contini & Pernin, 1975). Furthermore, the morphology of this ammonite, closer in shape with an exemplar found at the base of the *Couches d’Effingen*, than with the type exemplar found at the top (Enay, Contini & Boullier, 1988: p. 310), suggests an attribution of the *calcaires graveleux inférieurs* to the base of the *Bifurcatus* zone (see Enay, Contini & Boullier, 1988: fig. 6), i.e. to the *Stenocycloides* subzone (Table S2). This correlation is supported by ammonites collected in the basal part of the *Marnes de Besançon* (Table S2), which indicate the same biochronological attribution (Enay, Contini & Boullier, 1988). Such a scheme thus constrains the age of the Damparis fossils to the *Rotoides* or *Stenocycloides* subzones. In Switzerland, the dating of the Vorbourg Member is still debated between a stratigraphically correlated spatial grading with the base of the Günsberg Formation, dated from the *Grossouvrei* subzone (Gygi, 2012), and a new set of ammonites dating it to the *Stenocycloides* subzone (Comment & Ayer, 2010). The rejection of the *Rotoides* zone in these two schemes implies a more probable attribution of the *Tidalites de Mouchard* to the *Stenocycloides* zone. Nevertheless, it underlines the necessity to collect new ammonites precisely located throughout detailed profiles to solve the chronological attribution of the *Tidalites de Mouchard* Member at Damparis, in Franche-Comté, and at the scale of the platform. In addition, the absolute age of the Damparis fossils within the *Rotoides* or *Stenocycloides* subzones still needs to be estimated. Nevertheless, according to the recent scale of Cohen *et al*. (2013), a range between 163.5 ± 1.0 and 157.3 ± 1.0 Ma can be proposed, i.e. about 160 Ma old.

**Additional cyclostratigraphic correlations throughout the Damparis profile**

Concerning other third order cycle sequence boundaries, Ox7 is positioned at the top of the Hauptmumienbank Formation, correlated with the Geissberg Member (Gygi, 2012) and attributed to the *Berrense* subzone (Gygi, 2012: p. 27). Ox8 and Kim1 are respectively positioned in the middle and at the top of the Courgenay Formation in Switzerland (Gygi, Coe & Vail, 1998). At Damparis, the *marnes et calcaires* Formation terminates with a bed of marly limestone with ostreid shells coated by an onlitic crusting, overlain in the vicinity, as well as in a second core drilled southeast of the quarry, by a contrasting 3-m-thick grey-blue clayey limestone unit with a large population of *Zeillerina astartina* (Boullier, Contini & Pernin, 1975). The close resemblance of this succession with the marly Bure Member in Switzerland, formerly known as “*Humeralis* marls” (Gygi, 2000), and the underlying Hauptmumienbank Member whose oncolites formed around nerineid and ostreid shells (Gygi & Persoz, 1986), led to the placement of Ox 7 at the top of the oncolitic limestone. This chronological attribution is consistent with that of the upper part of the *Marnes de Besançon* dated by ammonites from the *Bimammatum* zone (Table S2; Enay, Contini & Boullier, 1988). In addition, Ox 7 is equivalent to the S2a–S2b lithoclinal sequence boundary of Enay, Contini & Boullier (1988) and Contini (1989), whereas the S1–S2 major boundary, located at the top of the *Calcaires de Clerval* Formation, matches with none of the other third order cycle sequence boundaries.

At the top of the Oxfordian sequence in the Dole vicinity, the *calcaires graveleux supérieurs* has a micritic texture, and its uppermost beds often show burrows, with the upper surface bored and covered by ostreid bivalves, which suggests its correlation with the *Calcaires de Besançon* encountered eastward in Franche-Comté and sharing the same characteristics (Enay, Contini & Boullier, 1988; Contini, 1989). Such a correlation is in agreement with that of Enay, Contini & Boullier (1988: p. 304, 324, figs 5, 6), who attributed all deposits above the *calcaires fins* to the *Marnes de Besançon*, which suggested a problematic correlation of Kimmeridgian limestones from Châtenois surroundings (Chauve *et al*., 1979) with the *Calcaires de Besançon*. Assuming that the *Calcaires de Besançon* is fully recorded by the *calcaires graveleux supérieurs*, sequence boundaries Ox 8 and Kim 1 would then respectively take place in the middle and at the top of this unit. The new correlation proposed here is thus more satisfying, but will require the local sampling of chronologically decisive ammonites to be fully demonstrated.

**PALAEOGEOGRAPHICAL IMPLICATIONS**

The succession of third order cycles Ox 5 to Ox 8 positioned here above throughout the Damparis profile encompasses both the late middle and upper Oxfordian (Fig. 7). It is centred on a second order regression that synchronously reached its maximum at Ox 6 in both Tethyan and Boreal domains (Hardenbol *et al*., 1998). This second order regression induced low eustatic levels and low maximum flooding surfaces from cycles Ox 3 to Ox 8 at a large scale, but also regionally, with northwest Switzerland characterised by a similar trend. Indeed, following a long term regression, the eustatic sea level reached a minimum that began before the *Grossouvrei* subzone and lasted to the top of the *Hauffianum* subzone, with little variation (Gygi, 2012: fig. 5.1). Consequently, ammonite faunas from both domains were separated until the upper Kimmeridgian (Enay, 1980), and important sedimentation gaps are associated with Ox 5 and, to a lesser extent, with Ox 6 to Ox 8 in synthetic cyclostratigraphic frameworks (Fig.  7). Locally, unconformities also punctuate the upper Oxfordian record on the Jura platform and may be associated with wood debris (Enay, Contini & Boullier, 1988; Lathuilière *et al*., 2005; Strasser, 2007; Comment & Ayer, 2010). The best example is provided by evidence of the *Brachyphylum* taxa and other plant remains in the basal marl lenses and limestone beds of the *Calcaires de l’Isle-sur-le-Doubs* (Fig.  7).

Garcia, Philippe & Gaumet (1998) indeed showed that carbonated platforms from the Bathonian to the Oxfordian were colonised by *Brachyoxylon*-dominated pioneer fauna during short emersion phases. During the subsequent flooding of the carbonate platform, wood remains were thus buried at a short distance from where vegetation developed. A similar process led to the preservation of the Damparis dinosaurs soon after Ox 5, and of several sauropod and theropod trackways in the Jura Mountains, all preserved during upper Oxfordian cyclical progradation phases of the carbonate platform (Cariou *et al*., 2014). The presence of such large animals implies that dense and rich vegetation must have been around to feed them during these episodes. For such a reason, the Damparis fossil assemblage is thus considered as evidence for emersion not only locally, but also possibly of large extension during a short emersion (Enay, 1980; Buffetaut, 1988).

To explain the megatracksite of Loulle (Jura, France), located about 50 km to the southeast of Damparis and recorded 3–2 myr later during the *Bimammatum* zone, Cariou *et al*. (2014) envisaged migrations of dinosaurs along an ENE-WSW exposed isthmus formed by contemporaneous emersions. In the case of Damparis, the site occupies a backward position on the platform, which was less extended at the end of the *Transversarium* zone (Enay, Contini & Boullier, 1988). Moreover, the evidence for emersion compiled from the almost contemporaneous *Calcaire de l’Isle-sur-le-Doubs* Member appears much more scattered in the middle of the platform edge (Fig. S1). Such a spatial distribution of this evidence, combined with the horizontal position of both wood debris and associated molluscs parallel to the stratification in the shallow depositional environments of this member (Enay, Contini & Boullier, 1988, Contini, 1989), led Contini (1972) to envisage a quiet marine depositional context between flat islets resulting from the emersion of prior bioherms. Such a context would thus have favoured recurrent regional scale emersions of the platform, as well as dinosaur migrations around or across the platform at the *Transversarium*-*Bifurcatus* limit (Fig. S1), but also during the whole upper Oxfordian (Cariou *et al*., 2014).

**PHYLOGENETIC CHARACTER LIST**

Characters 1–397 are the same as those presented in previous iterations of the data matrix (Mannion *et al*., 2013; Poropat *et al*., 2015a,b, 2016; Upchurch, Mannion & Taylor, 2015), although one of these (character 40) is revised. Characters 398–416 are newly added to this data matrix.

C1. Premaxillary anterior margin, shape: without step or with anteroposteriorly short ‘muzzle’, less than 0.25 of skull length (measured up to the anterior point of the ascending process of premaxilla) (0); elongate, boot-shaped snout, equal to or greater than 0.25 of skull length (1) (Wilson, 2002; Mannion *et al*., 2013).

C2. External naris, greatest diameter to greatest diameter of orbit ratio: greater than 1.0 (0); 1.0 or less (1) (McIntosh, 1990; Upchurch, 1995; Upchurch *et al*., 2004; Mannion *et al*., 2013).

C3. Parietal occipital process, dorsoventral height to greatest diameter of foramen magnum ratio: greater than 1.0 (0); 1.0 or less (1) (Wilson, 2002; Mannion *et al*., 2013).

C4. Parietal, distance separating supratemporal fenestrae to long axis of supratemporal fenestra ratio: 1.0 or greater (0); less than 1.0 (1) (Wilson, 2002; Mannion *et al*., 2013).

C5. Quadratojugal, anterior process to dorsal process length ratio: 1.3 or less (0); greater than 1.3 (1) (Upchurch, 1998; Wilson & Sereno, 1998; Mannion *et al*., 2013).

C6. Supraoccipital, dorsoventral height to foramen magnum dorsoventral height ratio: 1.0 or greater (0); less than 1.0 (1) (Wilson, 2002; Mannion *et al*., 2013).

C7. Occipital condyle, dorsoventral height to combined occipital condyle + basal tubera dorsoventral height ratio: less than 0.6 (0); 0.6 or greater (1) (Mannion, 2011; Mannion *et al*., 2013).

C8. Basal tubera, mediolateral width to occipital condyle mediolateral width: less than 1.5 (0); 1.5 or greater (1) (Wilson, 2002; Mannion, 2011; Mannion *et al*., 2013).

C9. Basipterygoid processes, length to basal diameter ratio: less than 3.0 (0); 3.0 or greater (1) (Wilson, 2002; Upchurch *et al*., 2004; Mannion *et al*., 2013; length is measured up to the base of the basal tubera).

C10. Surangular, dorsoventral height to maximum dorsoventral height of angular ratio: 2.0 or greater (0); less than 2.0 (1) (Wilson & Sereno, 1998; Upchurch *et al*., 2004; Mannion *et al*., 2013).

C11. Tooth crowns, Slenderness Index (SI) values (apicobasal length of the tooth crown divided by its maximum mesiodistal width): less than 2.0 (0); 2.0 to <4.0 (1); 4.0 or greater (2) (Upchurch, 1998; Upchurch *et al*., 2004; Mannion *et al*., 2013) [ordered].

C12. Maxillary teeth, number: 17 or more (0); fewer than 17 (1) (Mannion *et al*., 2013).

C13. Dentary teeth, number: greater than 15 (0); 15 or fewer (1) (Wilson & Sereno, 1998; Mannion *et al*., 2013).

C14. Cervical vertebrae, number: 13 or fewer (0); 14–15 (1); more than 15 (2) (Upchurch, 1995, 1998; Wilson & Sereno, 1998; Mannion *et al*., 2013) [ordered].

C15. Cervical centra, highest average Elongation Index value (aEI; centrum anteroposterior length [excluding articular ball] divided by the mean average value of the posterior articular surface mediolateral width and dorsoventral height) of: less than 3.0 (0); between 3.0 and <4.0 (1); greater than 4.0 (2) (Upchurch, 1995, 1998; Upchurch *et al*., 2004; Chure *et al*., 2010) [ordered].

C16. Anterior cervical centra, posterior articular face dorsoventral height to mediolateral width ratio: 1.0 or greater (0); less than 1.0 (1) (Upchurch, 1998; Upchurch *et al*., 2004; Mannion *et al*., 2013).

C17. Middle–posterior cervical centra, posterior articular face dorsoventral height to mediolateral width ratio: 1.0 or less (0); greater than 1.0 (1) (Curry Rogers, 2005; Mannion *et al*., 2013).

C18. Posterior cervical neural arch to centrum dorsoventral height ratio, measured on anterior face of vertebra (arch height measured from dorsal surface of centrum to base of prezygapophyses): 0.5 or greater (0); less than 0.5 (1) (Bonaparte *et al*., 2006; Mannion *et al*., 2013).

C19. Posteriormost cervical and anteriormost dorsal neural spines, dorsoventral height divided by posterior centrum height: 1.0 or greater (0); less than 1.0 (1) (D’Emic, 2012; Mannion *et al*., 2013).

C20. Dorsal vertebrae, number: 13 or more (0); 12 or fewer (1) (McIntosh, 1990; Upchurch, 1998; Wilson & Sereno, 1998; Upchurch *et al*., 2004).

C21. Anterior dorsal centra, posterior articular face mediolateral width to dorsoventral height ratio: less than 1.3 (0); 1.3 or greater (1) (Mannion *et al*., 2013).

C22. Middle–posterior dorsal centra, posterior articular face mediolateral width to dorsoventral height ratio: less than 1.0 (0); 1.0 or greater (1) (Upchurch, 1998; Upchurch *et al*., 2004; Mannion *et al*., 2013).

C23. Posterior dorsal neural spines, dorsoventral height divided by posterior centrum dorsoventral height: 1.0 or greater (0); less than 1.0 (1) (McIntosh, 1990; Upchurch, 1995, 1998; Mannion *et al*., 2013).

C24. Sacral vertebrae, number: 5 or fewer (0); 6 or more (1) (McIntosh, 1990; Upchurch, 1995, 1998; Wilson & Sereno, 1998).

C25. Anterior caudal centra, mediolateral width to dorsoventral height (excluding chevron facets) of anterior surface ratio: less than 1.0 (0); 1.0 or greater (1) (Upchurch *et al*., 2004; Mannion *et al*., 2013).

C26. Anterior caudal centra, lowest average Elongation Index (aEI; centrum anteroposterior length [excluding articular ball] divided by the mean average value of the anterior surface mediolateral width and dorsoventral height) value of: less than 0.6 (0); 0.6 or greater (1) (Gauthier, 1986; Upchurch, 1995, 1998; Upchurch *et al*., 2004; Mannion *et al*., 2013).

C27. Anterior caudal centra, anteroposterior length of posterior condylar ball to mean average radius ([mediolateral width + dorsoventral height] divided by 4) of anterior articular surface of centrum ratio: zero (posterior articular surface of centrum is flat or concave) (0); less than or equal to 0.3 (posterior articular surface of centrum is mildly convex) (1); greater than 0.3 (posterior articular surface of centrum is strongly convex) (2) (McIntosh, 1990; Upchurch, 1995, 1998; Salgado *et al*., 1997; Wilson, 2002; Whitlock *et al*., 2011; Mannion *et al*., 2013; note that the highest value for a taxon is always used) [ordered].

C28. Middle caudal centra, mediolateral width to dorsoventral height (excluding chevron facets) of anterior surface ratio: less than 1.0 (0); 1.0 or greater (1) (Upchurch *et al*., 2004; Mannion *et al*., 2013).

C29. Middle caudal centra, average Elongation Index (aEI; centrum anteroposterior length [excluding articular ball] divided by the mean average value of the anterior surface mediolateral width and dorsoventral height [excluding chevron facets]) value: less than 1.4 (0); 1.4 or higher (1) (Upchurch & Martin, 2003; Upchurch *et al*., 2004; Whitlock, 2011; Mannion *et al*., 2013).

C30. Posterior caudal centra, mediolateral width to dorsoventral height (excluding chevron facets) of anterior surface ratio: less than 1.2 (0); 1.2 or greater (1) (Upchurch *et al*., 2004; Mannion *et al*., 2013).

C31. Posterior caudal centra, average Elongation Index (aEI; centrum anteroposterior length [excluding articular ball] divided by the mean average value of the anterior surface mediolateral width and dorsoventral height [excluding chevron facets]) value: less than 1.7 (0); 1.7 or higher (1) (Upchurch & Martin, 2003; Upchurch *et al*., 2004; Whitlock, 2011; Mannion *et al*., 2013).

C32. Anteriormost caudal neural spines, dorsoventral height divided by centrum height: 1.2 or greater (0); less than 1.2 (1) (McIntosh, 1990; Calvo & Salgado, 1995; Upchurch, 1995, 1998; Mannion *et al*., 2013).

C33. Anterior caudal neural spines, maximum mediolateral width to anteroposterior length ratio: less than 1.0 (0); 1.0 or greater (1) (Upchurch, 1998; Mannion *et al*., 2013).

C34. Anterior caudal neural spines, maximum mediolateral width to minimum mediolateral width ratio: less than 2.0 (0); 2.0 or greater (spines expand dorsally, forming ‘club’- or ‘mace’-shaped spinous processes) (1) (Canudo *et al*., 2008; Taylor, 2009; Mannion *et al*., 2013).

C35. Anterior chevrons (excluding first chevron), dorsoventral height of haemal canal divided by total chevron height: less than 0.40 (0); 0.40 or greater (1) (Curry Rogers & Forster, 2001; Wilson, 2002; Mannion *et al*., 2013; note that dorsoventral height of the haemal canal is measured from the proximal tip of the chevron down to the distal tip of the haemal canal, regardless of whether the chevron is dorsally bridged).

C36. Scapular acromion process, dorsoventral height to minimum dorsoventral height of scapular blade ratio: less than 3.0 (0); 3.0 or greater (1) (Wilson & Sereno, 1998; Mannion *et al*., 2013; dorsoventral height is measured perpendicular to long-axis of scapular blade).

C37. Scapular blade, maximum (measured at or close to distal end) to minimum dorsoventral height ratio: 2.0 or greater (0); less than 2.0 (1) (Wilson, 2002; Rose, 2007; Mannion *et al*., 2013).

C38. Coracoid, anteroposterior length to dorsoventral height of scapular articulation ratio: 1.0 or greater (0); less than 1.0 (1) (Wilson, 2002; Mannion *et al*., 2013).

C39. Sternal plate, maximum length divided by humerus proximodistal length: less than 0.65 (0); 0.65 or greater (1) (McIntosh, 1990; Upchurch, 1998; Mannion *et al*., 2013).

C40. Humerus to femur proximodistal length ratio: 0.8 or less (0); >0.8 to <0.9 (1); 0.9–0.95 (2); >0.95 (3) (Wilson, 2002; Upchurch *et al*., 2004; Poropat *et al*., 2016; an additional state has been added here to capture variation within Titanosauriformes) [ordered].

C41. Humerus, maximum mediolateral width of proximal end divided by proximodistal length: 0.4 or greater (0); less than 0.4 (1) (Mannion *et al*., 2013).

C42. Humerus, minimum mediolateral width divided by proximodistal length: 0.15 or greater (0); less than 0.15 (1) (Curry Rogers, 2005; Mannion *et al*., 2013).

C43. Humerus shaft eccentricity, mediolateral to anteroposterior width ratio at midshaft: greater than 1.5 (usually close to 1.8) (0); 1.5 or lower (usually close to 1.3) (1) (Wilson, 2002; Mannion *et al*., 2012, 2013).

C44. Radius to humerus proximodistal length ratio: 0.65 or greater (0); less than 0.65 (1) (Yates & Kitching, 2003; Mannion *et al*., 2013).

C45. Radius, maximum diameter of the proximal end divided by proximodistal length: less than 0.3 (0); 0.3 or greater (1) (McIntosh, 1990; Upchurch, 1995, 1998; Upchurch *et al*., 2004).

C46. Radius, mediolateral width of proximal to distal end ratio: 1.0 or greater (0); less than 1.0 (1) (Curry Rogers, 2005; Mannion *et al*., 2013; note that in taxa with a twisted radius, the dimension of the long axis of the distal end is used).

C47. Radius, distal end mediolateral width to midshaft mediolateral width ratio: less than 2.0 (0); 2.0 or greater (1) (Wilson, 2002; Rose, 2007; Mannion *et al*., 2013; note that in taxa with a twisted radius, the dimension of the long axis of the distal end is used).

C48. Radius, distal end mediolateral to anteroposterior width ratio: 1.5 or greater (0); less than 1.5 (1) (Wilson & Sereno, 1998; Mannion *et al*., 2013).

C49. Radius, distal condyle orientation: perpendicular or bevelled less than 20° to long axis of shaft (0); bevelled at least 20° to long axis of shaft (1) (Curry Rogers & Forster, 2001; Wilson, 2002; Mannion *et al*., 2013; note that in most taxa only the lateral half of the distal end is bevelled, but this is used as the measurement in those instances).

C50. Ulna, ratio of maximum mediolateral width of proximal end to ulna length: gracile, ratio is less than 0.4 (0); stout, ratio is 0.4 or greater (1) (Wilson, 2002; Curry Rogers, 2005; Mannion *et al*., 2013).

C51. Ulna, ratio of mediolateral width of proximal end (equivalent to anteromedial arm) to anteroposterior width of proximal end (equivalent to anterolateral arm): less than 1.4 (0); 1.4 to <2.0 (1); 2.0 or greater (2) (Wilson 2002; Mannion *et al*. 2013; Upchurch *et al*. 2015; the long-axes of the anteromedial and anterolateral processes are extrapolated posteriorly so that they intersect close to the position of the olecranon, and each process length is then measured from this intersection to the tip of each process) [ordered].

C52. Metacarpals, longest metacarpal to radius proximodistal length ratio: less than 0.40 (0); 0.40 or greater (1) (McIntosh, 1990; Calvo & Salgado, 1995; Upchurch, 1998; Wilson & Sereno, 1998; Upchurch *et al*., 2004).

C53. Metacarpals, metacarpal I proximal end dorsoventral height to mediolateral width ratio: less than 1.8 (0); 1.8 or greater (1) (Apesteguía, 2005a; Mannion & Calvo, 2011; Mannion *et al*., 2013; note that the metacarpal is measured with the flat surface of the ‘D’ shape facing laterally, such that the long axis is dorsoventrally aligned).

C54. Metacarpals, metacarpal I to metacarpal II or III proximodistal length ratio: less than 1.0 (0); 1.0 or greater (1) (Upchurch, 1998; note that an average is taken when both metacarpals II and III are preserved).

C55. Metacarpals, metacarpal I to metacarpal IV proximodistal length ratio: less than 1.0 (0); 1.0 or greater (1) (Wilson & Sereno, 1998).

C56. Manual ungual on digit I to metacarpal I proximodistal length ratio: 0.5 or greater (0); less than 0.5 (1) (Upchurch *et al*., 2004; Mannion *et al*., 2013).

C57. Ilium, pubic peduncle (measured at the articular surface), anteroposterior to mediolateral width ratio: greater than 0.5 (0); 0.5 or less (1) (Taylor, 2009; Mannion *et al*., 2013).

C58. Pubis, iliac articular surface, anteroposterior to mediolateral width ratio: less than 2.0 (0); 2.0 or greater (1) (Mannion & Calvo, 2011; Mannion *et al*., 2013).

C59. Pubis, dorsoventral height of ischial articulation of the pubis divided by pubis proximodistal length is: 0.4 or greater (0); less than 0.4 (1) (Salgado *et al*., 1997; Wilson & Sereno, 1998; Upchurch *et al*., 2004; Mannion *et al*., 2013).

C60. Ischium to pubis proximodistal length ratio: greater than 0.8 (0); 0.80 or less (1) (Calvo & Salgado, 1995; Salgado *et al*., 1997; Upchurch, 1998; Mannion *et al*., 2013).

C61. Ischium, ratio of anteroposterior length of proximal plate to ischium proximodistal length: greater than 0.25 (0); 0.25 or less (1) (Mannion *et al*., 2013).

C62. Ischium, ratio of anteroposterior length of iliac peduncle to anteroposterior length of proximal plate: less than 0.7 (large ischial contribution to acetabulum) (0); 0.7 or greater (small ischial contribution to acetabulum) (1) (Wilson, 2002; D’Emic, 2012; Mannion *et al*., 2013).

C63. Ischium, ratio of dorsoventral width across the distal shaft (mediolateral in taxa with fully coplanar shafts) to ischium proximodistal length: 0.2 or greater (0); less than 0.2 (1) (Jacobs *et al*., 1993; Upchurch, 1998; Upchurch *et al*., 2004; Mannion *et al*., 2013).

C64. Ischium, ratio of dorsoventral width of distal end of shaft to minimum shaft dorsoventral width (both dimensions are mediolateral in taxa with fully coplanar shafts): 1.5 or greater (0); less than 1.5 (1) (Berman & McIntosh, 1978; McIntosh, 1990; Upchurch, 1995, 1998; Mannion *et al*., 2013).

C65. Femur shaft eccentricity, mediolateral width to anteroposterior width ratio at midshaft: less than 1.85 (0); 1.85 or greater (1) (Wilson, 2002; Mannion *et al*., 2013).

C66. Femoral distal condyles, tibial to fibular condylar anteroposterior length ratio: less than 1.2 (0); 1.2 or greater (1) (Upchurch *et al*., 2004; Mannion *et al*., 2013).

C67. Tibia, distal end mediolateral width to long-axis of a cross section horizontally through the midshaft ratio: 2.0 or greater (0); less than 2.0 (1) (Wilson, 2002; Mannion *et al*., 2013).

C68. Tibia, distal end, mediolateral to anteroposterior width ratio: 1.5 or greater (0); less than 1.5 (1) (Salgado *et al*., 1997; Upchurch *et al*., 2004; Mannion *et al*., 2013).

C69. Fibula, mediolateral width of distal end to mediolateral width at midshaft ratio: 2.0 or greater (0); less than 2.0 (1) (Wilson, 2002; Mannion *et al*., 2013).

C70. Astragalus, mediolateral width to maximum proximodistal height ratio: 1.8 or greater (0); less than 1.8 (1) (Wilson, 2002; Mannion *et al*., 2013).

C71. Astragalus, mediolateral width to maximum anteroposterior length ratio: 1.5 or greater (0); less than 1.5 (1) (D’Emic, 2012; Mannion *et al*., 2013).

C72. Metatarsals, metatarsal I to metatarsal V proximodistal length ratio: less than 1.0 (0); 1.0 or greater (1) (Mannion *et al*., 2013).

C73. Metatarsals, metatarsal III to tibia proximodistal length ratio: less than 0.25 (0); 0.25 or greater (1) (Wilson & Sereno, 1998; Upchurch *et al*., 2004; Mannion *et al*., 2013).

C74. Metatarsals, metatarsal V proximal end to distal end maximum mediolateral width ratio: 1.6 or greater (0); less than 1.6 (1) (Mannion *et al*., 2013).

C75. Premaxilla, posterolateral processes and lateral processes of maxilla: without midline contact (0); with midline contact forming marked narial depression, subnarial foramen not visible laterally (1) (Upchurch, 1998; Wilson & Sereno, 1998).

C76. Premaxillary anterior margin, shape: with step (0); without step (1) (Upchurch, 1995, 1998; Wilson & Sereno, 1998; Mannion *et al*., 2013).

C77. Premaxilla–maxilla sutural contact, shape in lateral view: straight (0); sinuous (1) (Chure *et al*., 2010).

C78. Maxillary ascending process, medial plate-like projections: do not contact each other on the midline (0); contact each other on the midline (1) (Upchurch, 1998).

C79. Maxilla, preantorbital fenestra: absent (0); present (1) (Berman & McIntosh, 1978; Upchurch, 1995, 1998; Wilson & Sereno, 1998).

C80. Lacrimal, anterior process: absent (0); present (1) (Wilson, 2002; polarity reversed here).

C81. Jugal–quadratojugal contact: articulation point includes the posterior margin of jugal (0); posterior margin of jugal excluded from articulation and only the ventral margin of the jugal contributes to articulation (1) (Curry Rogers, 2005; Mannion *et al*., 2013).

C82. Prefrontal, shape of posterior end in dorsal view: acute, with a subtriangular outline (0); broadly rounded or ‘square’ (1) (Berman & McIntosh, 1978; Upchurch, 1998; Mannion *et al*., 2013).

C83. Frontal, medial convexity in dorsal view: absent (0); present (1) (Curry Rogers, 2005).

C84. Parietal, elongate posterolateral process: present (0); absent (1) (Curry Rogers, 2005; Mannion *et al*., 2013).

C85. Parietal, contribution to post-temporal fenestra: present (0); absent (1) (Wilson, 2002).

C86. Supratemporal fenestra, lateral exposure: visible laterally, temporal bar shifted ventrally (0); not visible laterally, obscured by temporal bar (1) (Wilson & Sereno, 1998; Mannion *et al*., 2013).

C87. Postorbital, ventral process: anteroposterior and mediolateral diameters equal, or mediolaterally compressed (0); anteroposteriorly compressed (1) (Wilson & Sereno, 1998; Upchurch *et al*., 2004).

C88. Infratemporal (or laterotemporal) fenestra, anterior extension: reaching midpoint of orbit (0); reaching or surpassing anterior margin of orbit (1) (Upchurch, 1995, 1998; Ksepka & Norell, 2010; Mannion *et al*., 2013).

C89. Squamosal–quadratojugal contact: present (0); absent (1) (Gauthier, 1986; Upchurch, 1995, 1998; Wilson & Sereno, 1998).

C90. Quadratojugal, anterior ramus, ventral triangular projection (close to the anterior tip): absent (0); present (1) (D’Emic, 2012).

C91. Quadrate, excavation in the posterior surface: absent or shallow (0); deep (1) (Wilson & Sereno, 1998; Upchurch *et al*., 2004).

C92. Quadrate fossa, orientation: posterior (0); posterolateral (1) (Wilson, 2002).

C93. Palatobasal contact for basipterygoid articulation has a dorsomedially oriented ‘hook’- or ‘finger’-like projection which curves round to clasp the end of the basipterygoid process: present (0); absent (1) (Madsen *et al*., 1995; Upchurch, 1998; Wilson & Sereno, 1998; Wilson, 2002; Mannion *et al*., 2013).

C94. Palatine, dorsomedial blade (that articulates with maxilla), lateral margin: curved (0); straight (1) (Wilson & Upchurch, 2009; Mannion *et al*., 2013).

C95. Vomer, anterior articulation with: maxilla (0); premaxilla (1) (Wilson, 2002).

C96. Paroccipital process, ventral non-articular process: absent (0); present (1) (Wilson, 2002).

C97. Basal tubera, degree of divergence: no divergence, or restricted to ventral half of basal tubera (0); extends into dorsal half of basal tubera, usually fully divergent (1) (Curry Rogers, 2005; Mannion, 2011; Mannion *et al*., 2013).

C98. Basioccipital, fossa/fossae on the posterior surface of the basal tubera: absent (0); present (1) (Wilson, 2002; Mannion, 2011; Mannion *et al*., 2013).

C99. Basioccipital, foramen/foramina between basal tubera and basipterygoid processes: present (0); absent (1) (Wilson, 2002; Mannion, 2011; Mannion *et al*., 2013).

C100. Basisphenoid–quadrate contact: absent (0); present (posterior surface of basal tubera bordered laterally and ventrally by a raised lip) (1) (Wilson, 2002, 2005).

C101. Basipterygoid processes, shape in cross-section: elliptical or subtriangular (0); subcircular (1) (Upchurch *et al*., 2004).

C102. External mandibular fenestra: present (0); absent (1) (McIntosh, 1990; Upchurch, 1995).

C103. Dentary, posteroventral process, shape: single (0); forked (1) (Chure *et al*., 2010).

C104. Tooth rows: restricted anterior to orbit (0); restricted anterior to antorbital fenestra (1); restricted anterior to external naris (2); restricted anterior to subnarial foramen (3) (Gauthier, 1986; Upchurch, 1998; Wilson, 2002; Chure *et al*., 2010; Mannion *et al*., 2013) [ordered].

C105. Teeth, occlusal (wear) pattern: interlocking, V-shaped facets (0); high angled planar facets (1); low angled planar facets (2) (Wilson & Sereno, 1998).

C106. Tooth crowns, orientation: aligned anterolingually, tooth crowns overlap (0); aligned along jaw axis, crowns do not overlap (1) (Wilson, 2002; Mannion *et al*., 2013).

C107. Tooth crowns in upper and lower tooth rows, relative diameters: subequal (0); lower crowns smaller than upper crowns (1) (Chure *et al*., 2010).

C108. Tooth crowns, shape in labial view: spatulate or ‘spoon’-like (i.e. constricted at the base relative to midheight of the crown) (0); parallel-sided (i.e. little expansion above the root) (1) (Calvo, 1994; Upchurch, 1998).

C109. Tooth crowns, cross-sectional shape at mid-crown: ‘D’-shaped (0); cylindrical (1) (Wilson & Sereno, 1998; Mannion *et al*., 2013).

C110. Tooth crowns, lingual surface: concave or flat (0); convex (1) (Upchurch, 1998; Upchurch *et al*., 2004; Mannion *et al*., 2013).

C111. Tooth crowns, apicobasally oriented lingual ridge: present (0); absent (1) (Barrett *et al*., 2002; Mannion *et al*., 2013).

C112. Tooth crowns, distinct mesial and distal carinae (labiolingually thinner than the rest of the tooth crown) along the full crown length: absent (0); present (1) (Mannion, 2011; Mannion *et al*., 2013).

C113. Tooth serrations/denticles: present (0); absent (1) (Wilson, 2002; Upchurch *et al*., 2004; Mannion *et al*., 2013).

C114. Maxillary teeth, shape: straight along axis (0); twisted axially through an arc of 30-45 degrees (1) (Chure *et al*., 2010; D’Emic, 2012).

C115. Cervical and anteriormost dorsal vertebrae, internal tissue structure: solid (0); camerate (1); camellate (2) (Wilson & Sereno, 1998; Carballido *et al*., 2011; Mannion *et al*., 2013).

C116. Atlantal intercentrum, occipital facet shape: rectangular in lateral view, length of dorsal aspect subequal to that of ventral aspect (0); expanded anteroventrally in lateral view, anteroposterior length of dorsal aspect shorter than that of ventral aspect, producing an anteroventral lip (1) (Wilson, 2002).

C117. Cervical axis, midline ventral keel: absent (0); present (1) (Mannion, 2011; Mannion *et al*., 2013).

C118. Postaxial cervical centra, anterior half of ventral surfaces are: flat or mildly convex mediolaterally (0); concave mediolaterally (1) (Upchurch, 1998; Mannion *et al*., 2013).

C119. Postaxial cervical centra, posterior half of ventral surfaces are: flat or mildly convex mediolaterally (0); concave mediolaterally (1) (Upchurch, 1998; Mannion *et al*., 2013).

C120. Postaxial cervical centra, ventral midline keel: present (0); absent (1) (Upchurch, 1998; Mannion *et al*., 2013).

C121. Postaxial cervical centra, parapophyses dorsally excavated: absent (0); present (1) (Upchurch, 1998; Mannion *et al*., 2013).

C122. Postaxial cervical centra, lateral surfaces: lack an excavation or have a shallow fossa (0); possess a deep foramen that is not divided into portions by accessory laminae (1); have a deep foramen that is divided into separate portions by one prominent and occasionally several smaller accessory laminae (2) (McIntosh, 1990; Russell & Zheng, 1993; Upchurch, 1995, 1998) [ordered].

C123. Middle cervical centra, lateral pneumatic fossa/foramen extends almost to the posterior end of the centrum, leaving only a thin strip of bone: absent (0); present (1) (Wedel *et al*., 2000; D’Emic, 2013; Mannion *et al*., 2013).

C124. Middle–posterior cervical centra, parapophyses, dorsal surfaces: face dorsally or slightly dorsolaterally (0); deflected to face strongly dorsolaterally, such that the cervical ribs are displaced ventrally at least the same height as the centrum (1) (Wilson & Upchurch, 2009; D’Emic, 2012; Mannion *et al*., 2013).

C125. Middle–posterior cervical centra, parapophyses: restricted to anterior half of centrum (excluding condylar ball) (0); elongate, extending more than half of the centrum length (excluding condylar ball) (D’Emic, 2012; Mannion *et al*., 2013).

C126. Cervical neural arches, ‘pre-epipophyses’ present on prezygapophyses: absent (0); present (1) (Wilson & Upchurch, 2009; Mannion *et al*., 2013).

C127. Cervical neural arches, epipophyses present on postzygapophyses: absent (0); present (1) (Yates, 2007).

C128. Cervical neural arches (post-Cv3), epipophyses: do not extend beyond the posterior margin of the postzygapophyses (0); extend beyond the posterior margin of the postzygapophyses (usually as prongs) (1) (Sereno *et al*., 1993; Yates, 2007; D’Emic, 2012; Mannion *et al*., 2013).

C129. Cervical neural arches, epipophyseal–prezygapophyseal lamina (EPRL): absent (0); present (1) (Sereno *et al*., 2007; Wilson & Upchurch, 2009).

C130. Middle–posterior cervical neural arches, centroprezygapophyseal lamina (CPRL): single (0); bifurcates into medial and lateral branches that both contact the prezygapophysis (1) (Upchurch, 1995, 1998; Wilson, 2002; Whitlock 2011a; Mannion *et al*., 2013).

C131. Middle–posterior cervical neural arches, intrapostzygapophyseal lamina (TPOL) projects beyond the posterior margin of the neural arch (including the centropostzygapophyseal laminae [CPOL]), forming a prominent subrectangular projection in lateral view: absent (0); present (1) (D’Emic, 2012; Mannion *et al*., 2013).

C132. Postaxial cervical and anterior dorsal neural spines: unbifurcated (0); bifurcated (1) (Gauthier, 1986; McIntosh, 1990; Upchurch, 1995, 1998; Wilson & Sereno, 1998).

C133. Cervical bifurcated neural spines (excluding the posteriormost cervical vertebrae), median process at base of ‘notch’: absent (0); present (1) (Gauthier, 1986; McIntosh, 1990; Upchurch, 1995, 1998; Wilson & Sereno, 1998; Mannion *et al*., 2013).

C134. Middle cervical neural spines, abrupt increase in height (height approximately doubled), following low anterior cervical neural spines (occurs around CV6-8): absent (0); present (1) (Wedel *et al*., 2000; Rose, 2007; Mannion *et al*., 2013).

C135. Middle cervical neural spines, dorsal surface with mediolaterally oriented midline ridge flanked by small fossae at its anterior and posterior ends: absent (0); present (1) (D’Emic, 2013; Mannion *et al*., 2013).

C136. Posterioriormost cervical and anterior dorsal neural arches, spinodiapophyseal lamina (SPDL): single structure (0); divided into anterior and posterior spinodiapophyseal laminae (1) (Salgado *et al*., 1997; Salgado & Powell, 2010; D’Emic, 2012).

C137. Posteriormost cervical and anterior dorsal unbifurcated neural spines, prespinal lamina: absent (0); present (1) (Salgado *et al*., 1997; D’Emic, 2012).

C138. Posteriormost cervical and anterior dorsal bifurcated neural spines, ‘trifid’ with median tubercle at least as tall as metapophyses: absent (0); present (1) (Wilson & Upchurch, 2009; D’Emic, 2012; Mannion *et al*., 2013).

C139. Cervical ribs, longest shafts are: short and do not project far beyond the end of the centrum to which they attach (0); elongate and form overlapping bundles (1) (McIntosh, 1990; Upchurch, 1995, 1998; Mannion *et al*., 2013).

C140. Cervical ribs, longest shafts extend beneath: fewer than 3 vertebrae (0); 3 vertebrae or more (1) (Wedel *et al*., 2000; Mannion *et al*., 2013).

C141. Middle–posterior dorsal vertebrae, internal tissue texture: solid (0); camerate (1); camellate (2) (Wilson & Sereno, 1998; Carballido *et al*., 2011; Mannion *et al*., 2013).

C142. Middle–posterior dorsal centra, ventral keel: absent (0); present (1) (Mannion *et al*., 2012; Poropat *et al*., 2016).

C143. Dorsal centra, lateral pneumatic foramen: absent (0); present (1) (Upchurch, 1998).

C144. Dorsal centra, lateral pneumatic foramina are: shallow fossae or excavations that do not ramify throughout the centrum (0); deep excavations that ramify throughout the centrum and into the base of the neural arch, often leaving only a thin septum on the midline of the centrum (1) (Upchurch, 1998; Mannion *et al*., 2013).

C145. Dorsal centra, lateral pneumatic foramina: have margins which are flush with the lateral surface of the centrum (0); are set within a lateral fossa on the lateral surface of the centrum (1) (Bonaparte & Coria, 1993; Upchurch *et al*., 2004).

C146. Anterior dorsal centra, lateral pneumatic foramina have: rounded posterior margins (0); acute posterior margins (1) (Upchurch, 1998).

C147. Middle–posterior dorsal centra, anterior articular face shape: flat or concave (0); mildly convex, with degree of convexity notably reducing along the dorsal sequence (1); strongly convex, with degree of convexity approximately consistent along the dorsal sequence (2) (Salgado *et al*., 1997; Wilson & Sereno, 1998; Mannion *et al*., 2013) [ordered].

C148. Middle–posterior dorsal neural arches, posterior centroparapophyseal lamina (PCPL): absent (0); present as a single lamina (1); two parallel laminae (2) (Upchurch, 1998; Wilson, 2002; D’Emic, 2012; Mannion *et al*., 2013) [ordered].

C149. Middle–posterior dorsal neural arches, hyposphene–hypantrum system: present (0); absent (1) (Salgado *et al*., 1997; Upchurch, 1998).

C150. Middle–posterior dorsal neural arches, hyposphene shape: narrow, ventral end subequal to or only slightly wider than dorsal tip (0); wide, triangular shape, with ventral end at least twice width of dorsal tip (1) (Apesteguía, 2005b; Mannion *et al*., 2013).

C151. Middle–posterior dorsal neural arches, posterior centrodiapophyseal lamina (PCDL): has an unexpanded ventral tip (0); expands and bifurcates towards its ventral tip (1) (Salgado *et al*., 1997).

C152. Middle–posterior dorsal neural arches, postzygodiapophyseal lamina (PODL): present (0); absent (1) (Salgado *et al*., 1997; Sanz *et al*., 1999; Mannion *et al*., 2013).

C153. Anterior dorsal diapophyses are: directed laterally or slightly upwards (0); directed strongly dorsolaterally at approximately 45° to the horizontal (1) (Upchurch, 1998; Mannion *et al*., 2013).

C154. Anterior–middle dorsal diapophyses: short and dorsoventrally tall (0); elongate and dorsoventrally narrow (1) (Janensch, 1950; Taylor, 2009; D’Emic, 2012).

C155. Middle–posterior dorsal diapophyses are: directed strongly dorsolaterally at approximately 45° to the horizontal (0); directed laterally or slightly upwards (1) (Upchurch, 1998; Mannion *et al*., 2013).

C156. Middle–posterior dorsal diapophyses, distal end: curves smoothly into the remaining dorsal surface of the process (0); is set off from the remaining dorsal surface by a lip, forming a distinct area (1) (Sanz *et al*., 1999; Upchurch *et al*., 2004; D’Emic, 2012).

C157. Posteriormost dorsal diapophyses lie: posterior or posterodorsal to the parapophysis (0); vertically above the parapophysis (1) (Upchurch, 1998).

C158. Dorsal neural spines, height: anterior dorsal neural spines subequal to or dorsoventrally shorter than posterior dorsal neural spines (0); anterior dorsal neural spines dorsoventrally taller than posterior dorsal neural spines (1) (Wilson, 2002; Taylor, 2009; Mannion *et al*., 2013).

C159. Dorsal neural spines, anteroposterior width: approximately constant along the height of the spine, with subparallel anterior and posterior margins (0); narrows dorsally to form a triangular shape in lateral view, with the base approximately twice the width as the dorsal tip (1) (Taylor, 2009; Mannion *et al*., 2013).

C160. Anterior dorsal neural spines, orientation: project dorsally or slightly anterodorsally (0); project posterodorsally (1) (Upchurch *et al*., 2004; Mannion *et al*., 2013).

C161. Middle dorsal neural spines: unbifurcated (0); bifurcated (dorsal surface excavated transversely) (1) (Mannion *et al*., 2013).

C162. Middle–posterior dorsal neural spines: tapering or not flaring distally (0); flared distally with triangular aliform processes projecting laterally from the top (formed primarily from the expansion of the spinopostzygapophyseal laminae [SPOLs]) (1) (Upchurch, 1998; Wilson & Sereno, 1998; Wilson, 2002; Upchurch *et al*., 2004).

C163. Middle–posterior dorsal neural spines: tapering or not flaring distally, or with absent or weakly developed triangular aliform processes (0); strongly developed triangular aliform processes so that the lateral tips of these processes extend further laterally than the postzygapophyses (1) (Upchurch, 1998; Wilson & Sereno, 1998; Wilson, 2002; Upchurch *et al*., 2004).

C164. Middle-posterior dorsal neural spines, orientation: vertical or slightly posterodorsal (0); strongly posterodorsal, oriented at 45° to the horizontal or greater (1) (Wilson 2002; Poropat *et al*., 2016; note that this excludes the posteriormost dorsal neural spines, which usually revert to being dorsally directed).

C165. Middle–posterior dorsal neural spines, spinopostzygapophyseal lamina (SPOL) shape: single (0); divided into medial and lateral branches (1) (Wilson, 1999, 2002).

C166. Middle–posterior dorsal neural spines, spinodiapophyseal lamina (SPDL): absent or restricted to posterior dorsals (0); present on middle and posterior dorsals (1) (Wilson, 2002; Upchurch *et al*., 2004).

C167. Middle–posterior dorsal neural spines, prespinal and postspinal laminae: form mediolaterally wide surfaces, with little anterior relief, ‘infilling’ the prespinal and postspinal fossae (0); form distinct mediolaterally narrow ridges or laminae along the midline of the prespinal and postspinal fossae (1) (Mannion *et al*., 2013).

C168. Middle–posterior dorsal neural spines, midline prespinal lamina (forming distinct ridge) along proximal (lower) half of neural spine: present (0); absent (1) (Salgado *et al*., 1997; Curry Rogers, 2005; Mannion *et al*., 2013; note that taxa coded as ‘0’ for C167 are scored as a ‘?’ here).

C169. Middle–posterior dorsal neural spines, postspinal lamina (forming distinct ridge) along proximal (lower) half of neural spine: present (0); absent (1) (Bonaparte, 1986; Upchurch, 1995, 1998; Curry Rogers, 2005; Mannion *et al*., 2013; note that taxa coded as ‘0’ for C167 are scored as a ‘?’ here).

C170. Thoracic (dorsal) ribs, pneumatised (with proximal pneumatocoels): absent (0); present (1) (Wilson & Sereno, 1998).

C171. Anterior thoracic ribs, cross-sectional shape: subcircular (0); plank-like, anteroposterior breadth more than three times mediolateral breadth (1) (Wilson, 2002).

C172. Sacral vertebrae, camellate internal tissue structure: absent (0); present (1) (Mannion *et al*., 2013).

C173. Sacral centra, lateral pneumatic foramina or very deep depressions: absent (0); present (1) (Upchurch, 1998).

C174. Sacral neural spines, dorsal portions of at least sacral vertebrae 1–4 fused, forming a dorsal ‘platform’: absent (0); present (1) (Lü *et al*., 2007; Mannion *et al*., 2013).

C175. Caudal vertebrae, number: more than 35 (0); 35 or fewer (1) (Wilson 2002; Mannion *et al*., 2013).

C176. Anteriormost caudal vertebrae, camellate internal tissue structure: absent (0); present (1) (Powell, 1986; Wilson, 2002).

C177. Anterior caudal centra, posterior articular surface: flat or concave throughout series (0); convex in anteriormost caudal vertebrae, changing to flat or concave in more distal anterior caudal vertebrae (1); convex throughout all anterior caudal vertebrae with ribs (2) (Mannion *et al*., 2013) [ordered].

C178. Anterior caudal centra, lateral pneumatic fossae or foramina: absent (0); present, (McIntosh, 1990; Upchurch, 1995).

C179. Anterior caudal centra, lateral pneumatic fossae or foramina: without sharply defined margins (0); with sharply defined margins (1) (Tidwell *et al*., 2001; Whitlock *et al*., 2011; Mannion *et al*., 2013).

C180. Anterior–middle caudal centra, small, shallow vascular foramina pierce the lateral and/or ventral surfaces: absent (0); present (1) (Mannion & Calvo, 2011; Mannion *et al*., 2013).

C181. Anterior–middle caudal centra (excluding the anteriormost caudal vertebrae), ventral longitudinal hollow: absent (0); present (1) (McIntosh, 1990; Upchurch, 1995, 1998; Wilson, 2002).

C182. Anterior–middle caudal centra (excluding the anteriormost caudal vertebrae), distinct ventrolateral ridges, extending the full length of the centrum: absent (0); present (1) (McIntosh, 1990; Upchurch, 1995, 1998; Upchurch *et al*., 2004; Mannion *et al*., 2013).

C183. Middle caudal centra, anteroposteriorly elongate ridge situated at approximately two-thirds of the way up the lateral surface: absent (0); present (1) (Upchurch & Martin, 2003; Mannion *et al*., 2013).

C184. Middle–posterior caudal centra (at least some), posterior articular surface: flat or concave (0); convex (1) (Jacobs *et al*., 1993; Upchurch, 1995, 1998; Salgado *et al*., 1997; Wilson, 2002).

C185. Middle–posterior caudal centra with convex posterior articular surface: condylar convexity merges smoothly into the lateral surface of the main body of the centrum (0); distinct rim rings the condyle, separating it from the lateral surface of the main body of the centrum (1) (Mannion *et al*., 2013).

C186. Distal caudal centra, biconvex: absent (0); present (1) (Wilson & Sereno, 1998).

C187. Anterior caudal neural arches, hyposphenal ridge: present (0); absent (1) (Upchurch, 1998; Mannion *et al*., 2013; usually only present in the anteriormost region of the tail).

C188. Anterior caudal neural arches, hyposphenal ridge shape: slender ridge (0); block-like hyposphene (1) (Taylor, 2009; Mannion *et al*., 2013).

C189. Anterior caudal neural arches, distinct prezygodiapophyseal lamina (PRDL): absent (0); present (1) (Chure *et al*., 2010; usually only present in the anteriormost region of the tail).

C190. Anterior caudal neural arches, sharp lipped lateral coel (postzygapophyseal centrodiapophyseal fossa [POCDF] of Wilson *et al*., 2011) bounded by posterior centrodiapophyseal lamina (PCDL [or caudal rib itself]), centropostzygapophyseal lamina (CPOL) and postzygodiapophyseal lamina (PODL): absent (0); present (1) (Lü *et al*., 2008; Whitlock *et al*., 2011; Mannion *et al*., 2013).

C191. Anterior–middle caudal neural arches, prezygapophyses switch from projecting anterodorsally, anteriorly and back to anterodorsally along the sequence: absent (0); present (1) (Mannion & Calvo, 2011; Mannion *et al*., 2013).

C192. Middle caudal neural arches: situated over the mid-point of the centrum with approximately subequal amounts of the centrum exposed at either end (0); located on the anterior half of the centrum (1) (Calvo & Salgado, 1995; Upchurch, 1995, 1998; Salgado *et al*., 1997).

C193. Middle–posterior caudal neural arches, distance that prezygapophyses extend beyond the anterior margin of the centrum: less than 20% of centrum length (excluding ball), short prezygapophyses (0); 20% or greater of centrum length (excluding ball), elongate prezygapophyses (1) (González Riga, 2003; Mannion *et al*., 2013; note that in taxa without anteriorly biased neural arches this prezygapophyseal extension is extrapolated as if the arch was anteriorly positioned on the centrum).

C194. Anteriormost caudal neural spines, sharp lipped lateral coel (spinodiapophyseal fossa [SDF] of Wilson *et al*., 2011) bounded by spinoprezygapophyseal lamina (SPRL), spinopostzygapophyseal lamina (SPOL) and postzygodiapophyseal lamina (PODL): absent (0); present (1) (Wilson, 2002; Whitlock *et al*., 2011; Wilson *et al*., 2011; Mannion *et al*., 2013).

C195. Anterior caudal neural spines, project: posterodorsally (0); dorsally (sometimes with a subtle anterior deflection) (1); anterodorsally, such that the anterodorsal margin of the neural spine projects beyond the anterior margin of the centrum (2) (Gonzalez Riga *et al*., 2009; Mannion *et al*., 2013; Poropat *et al*., 2016) [ordered].

C196. Anterior caudal neural spines, anterodorsal margin of neural spine: level with or posterior to posterior margin of postzygapophyses (0); situated anterior to posterior margin of postzygapophyses (usually does not even approach postzygapophyses) (1) (Salgado *et al*., 1997; Mannion *et al*., 2013).

C197. Anterior caudal neural spines, prespinal and postspinal laminae: absent or form mediolaterally wide surfaces, with little anterior relief, ‘infilling’ the prespinal and postspinal fossae (0); form distinct mediolaterally narrow ridges or laminae along the midline of the prespinal and postspinal fossae (1) (Mannion *et al*., 2013).

C198. Anterior caudal neural spines, spinoprezygapophyseal lamina (SPRL)–spinopostzygapophyseal lamina (SPOL) contact: absent (0); present, forming a prominent lateral lamina on the neural spine (1) (Wilson, 1999, 2002).

C199. Middle caudal neural spines, in lateral view, widen anteroposteriorly (approximately doubling) from their base to their summit: absent (0); present (1) (Mannion *et al*., 2013).

C200. Middle caudal neural spines, extend posteriorly to the mid-point (or beyond) of the proceeding caudal centrum: present (0); absent (usually do not extend beyond the posterior margin of the centrum) (1) (Remes *et al*., 2009; Mannion *et al*., 2013; note that in taxa with anteriorly biased neural arches this posterior extension is extrapolated as if the arch was centrally positioned on the centrum).

C201. Caudal ribs: present beyond approximately Cd10 (usually at least up to approximately Cd15) (0); only present through to approximately Cd10 (1) (Upchurch, 1998; Wilson, 2002).

C202. First caudal rib (transverse process), with prominent ventral bulge: absent (0); present (1) (Wilson, 2002; Chure *et al*., 2010; note that this feature is sometimes present in subsequent caudal vertebrae too: consequently, this character is coded as the derived state if present in any anterior caudal vertebrae, but coded as a ‘?’ if the first caudal vertebra is not preserved and the feature is absent in other anterior caudal vertebrae).

C203. First caudal rib, expands anteroposteriorly towards its distal end, forming an ‘anchor’ shape in dorsal view: absent (0); present (1) (Suteethorn *et al*., 2010; Mannion *et al*., 2013).

C204. Anterior caudal ribs, shape in anterior view: triangular, tapering distally (0); wing-like, with a dorsolaterally oriented dorsal margin (1) (Berman & McIntosh, 1978; McIntosh, 1990; Upchurch, 1995, 1998; Whitlock *et al*., 2011).

C205. Anterior caudal ribs: curve strongly anterolaterally (0); mainly laterally (1); curve strongly posterolaterally (2) (Mannion & Calvo, 2011; D’Emic, 2012; Mannion *et al*., 2013) [ordered].

C206. Anterior caudal ribs: do not extend beyond posterior end of centrum (excluding posterior ball) (0); extend beyond posterior end of centrum (excluding posterior ball) (1) (Mannion & Calvo, 2011; Mannion *et al*., 2013).

C207. First chevron, morphology: Y-shaped and does not differ notably from subsequent chevrons (0); anteroposteriorly flattened and V-shaped, with dorsoventrally reduced distal blade (1) (Gomani, 2005; Rauhut, 2006; Mannion & Calvo, 2011; Mannion *et al*., 2013).

C208. Anterior chevrons, proximal ends: open dorsally (0); bridged dorsally by a bar of bone (1) (Powell, 1992; Calvo & Salgado, 1995; Upchurch, 1995, 1998; Mannion *et al*., 2013).

C209. Anterior–middle chevrons, lateral bulges close to distal ends of chevron blades: absent (0); present (1) (Mannion *et al*., 2013).

C210. Middle–posterior chevrons, with anterior expansion of distal blade: present (0); absent (1) (Berman & McIntosh, 1978; Upchurch, 1995, 1998; Wilson & Sereno, 1998; Mannion *et al*., 2013).

C211. Middle–posterior chevrons, with posterior expansion of distal blade (excluding the natural posteroventral curvature of many chevrons): present (0); absent (1) (Berman & McIntosh, 1978; Upchurch, 1995, 1998; Wilson & Sereno, 1998; Mannion *et al*., 2013).

C212. Scapular acromion (proximal plate), area situated posterior to the acromial ridge: flat or convex (0); forms a separate excavated area (1) (Upchurch *et al*., 2004).

C213. Scapular glenoid surface, orientation: faces anteroventrally and/or slightly laterally (0); deflected to face anteroventrally and medially (1) (Wilson & Sereno, 1998; Upchurch *et al*., 2004).

C214. Scapula, posterior margin of the dorsal part of the acromion: straight and oriented vertically, or sloping to face posterodorsally (0); concave, posterodorsal corner of acromion overhangs the dorsal surface of the scapular blade (1) (Rauhut *et al*., 2005; Mannion, 2009; Mannion *et al*., 2013).

C215. Scapular acromion, subtriangular process at posteroventral corner: absent (0); present (1) (Smith *et al*., 2001; Bonaparte *et al*., 2006; Carballido *et al*., 2011; D’Emic *et al*., 2011; Mannion *et al*., 2013).

C216. Scapular blade, subtriangular process at anteroventral corner: absent (0); present (1) (Carballido *et al*., 2011; D’Emic *et al*., 2011; Mannion *et al*., 2013).

C217. Scapular blade, cross-sectional shape at base: rectangular (0); D-shaped (lateral surface is strongly convex dorsoventrally and medial surface flat) (1) (Wilson, 2002).

C218. Coracoid, anterior and dorsal margins in lateral view: merge smoothly into each other, giving a rounded profile (0); meet each other at an abrupt angle, making the coracoid quadrangular in outline (1) (Upchurch, 1998).

C219. Coracoid, dorsal margin in lateral view: lies below the level of the scapular acromion plate (separated from the latter by a V-shaped notch) (0); reaches or surpasses the level of the dorsal margin of the scapular acromion plate (1) (Upchurch, 1995, 1998; Upchurch *et al*., 2004; Mannion *et al*., 2013).

C220. Coracoid, ventral margin in lateral view forms a notch anterior to the glenoid, resulting in an ‘infraglenoid lip’ anterior to the notch: absent (0); present (1) (Wilson, 2002; Mannion *et al*., 2013).

C221. Sternal plate, shape in dorsal view: subcircular or oval (0); triangular (created by an acute anterolateral projection) (1); elliptical with a mildly or strongly concave lateral margin (2) (Calvo & Salgado, 1995; Upchurch, 1998; Wilson, 2002; Upchurch *et al*., 2004).

C222. Sternal plate, prominent posterolateral expansion produces a ‘kidney’-shaped profile in dorsal view: absent (0); present (1) (McIntosh, 1990; Upchurch, 1998).

C223. Humeral proximolateral corner, shape: rounded, surfaces merge smoothly into each other to produce a transversely rounded proximal end, with the proximalmost point of the lateral margin at a lower level than the remaining lateral half of the proximal surface (0); square, surfaces meet each other at an abrupt angle to produce a ‘squared’ proximal end in anterior view, with the proximalmost point of the lateral margin level with the remaining lateral half of the proximal surface (1) (Upchurch, 1999; Wilson, 2002; Upchurch *et al*., 2004; Harris, 2006; Mannion *et al*., 2013).

C224. Humerus, shape of lateral margin of diaphysis (approximately the middle third of the humerus) in anterior view: concave (0); straight (1) (Curry Rogers, 2005; Mannion *et al*., 2013).

C225. Humeral deltopectoral crest, projection: anteriorly or anterolaterally (0); anteromedially, extending across the anterior face of the humerus (1) (Mannion *et al*., 2013; Upchurch *et al*., 2015; Poropat *et al*., 2016).

C226. Humerus, strong bulge or tuberosity (site for M. latissimus dorsi) close to the lateral margin of the posterior surface, at approximately the level of the distal tip of the deltopectoral crest: absent (0); present (1) (Borsuk-Bialynicka, 1977; Otero, 2010; D’Emic, 2012).

C227. Humerus, anterior surface of distal lateral condyle: divided by a notch, forming two ridges (0); undivided (1) (D’Emic, 2012).

C228. Humerus, distalmost part of the posterior surface (supracondylar fossa) is: flat or shallowly concave (0); deeply concave between prominent lateral and medial vertical condylar ridges (1) (Upchurch *et al*., 2004).

C229. Humeral distal condyles, articular surface: flat anteroposteriorly and restricted to distal portion of humerus (0); anteroposteriorly convex so that it curves up onto the anterior and posterior faces of the humerus (1) (Wilson, 2002; Upchurch *et al*., 2004).

C230. Humeral distal articular surface, condyles: undivided (0); divided (1) (Wilson, 2002; Mannion *et al*., 2013).

C231. Radius, strong twist in axis, such that the long-axes of the proximal and distal ends are not oriented in the same plane: absent (0); present (1) (Mannion *et al*., 2013).

C232. Radius, well developed interosseous ridge that extends along most of the radius length (at least along the distal two-thirds): absent (0); present (1) (Curry Rogers, 2005; Mannion *et al*., 2013).

C233. Ulnar olecranon process, development: absent or only rudimentary, i.e. projecting just above the proximal articulation (0); prominent, projecting well above proximal articulation (1) (McIntosh, 1990; Wilson & Sereno, 1998; Mannion *et al*., 2013).

C234. Ulna, articular surface of anteromedial process is: flat (0); concave along its length (1) (Upchurch, 1995, 1998).

C235. Ulna, orientation of anteromedial process: flat or sloping downwards less than 40° (0); sloping downwards at an angle of at least 40° to the horizontal (1) (Mannion *et al*., 2013).

C236. Ulna, distal end: prominently expanded posteriorly (0); unexpanded (1) (D’Emic, 2012).

C237. Carpal bones, number: 3 or more (0); fewer than 3 (1) (Upchurch, 1995, 1998).

C238. Carpal bones: at least one carpal present (0); absent (1) (Upchurch, 1995, 1998).

C239. Metacarpals, distal articular surfaces: extend onto dorsal/anterior surface of metacarpal (0); restricted to distal surface (except sometimes in metacarpal IV) (1) (Salgado *et al*., 1997; D’Emic, 2012).

C240. Metacarpals, metacarpal I distal end mediolateral axis orientation: approximately perpendicular (or only gently bevelled) to long axis of shaft (0); bevelled approximately 20° proximodistally with respect to axis of shaft (1) (Wilson, 2002; Mannion *et al*., 2013).

C241. Metacarpals, metacarpal IV has a prominent proximolateral projection that wraps around the dorsal (anterior) face of metacarpal V (metacarpal IV often forms a chevron shape in proximal end view): absent (0); present (1) (D’Emic, 2012; Mannion *et al*., 2013).

C242. Manual digits: possess at least some phalanges (0); have lost the phalanges (1) (Wilson, 2002; Upchurch *et al*., 2004).

C243. Manual phalanx I.1, shape in dorsal view: rectangular (0); wedge-shaped (1) (Wilson, 2002).

C244. Ilium, preacetabular process in dorsal view: projects anteriorly (0); projects anterolaterally (1) (Upchurch *et al*., 2004).

C245. Ilium, preacetabular process orientation: lies in an approximately vertical plane (0); turns laterally towards its ventral tip to form a horizontal portion (1) (McIntosh, 1990; Powell, 1992; Upchurch, 1995, 1998).

C246. Ilium, preacetabular process shape: dorsoventrally tapers anteriorly to a point (0); semicircular, or rounded outline, such that it does not continue to taper along its anteriormost portion (1) (Calvo & Salgado, 1995; Upchurch, 1998; Wilson & Sereno, 1998; Mannion *et al*., 2013).

C247. Ilium, preacetabular process, bulge or ‘kink’ on ventral margin: absent (0); present (1) (D’Emic, 2012).

C248. Ilium, highest point on the dorsal margin: occurs level with or posterior to the anterior margin of the base of the pubic process (0); occurs anterior to the anterior margin of the base of the pubic process (1) (Upchurch, 1998; Mannion *et al*., 2013).

C249. Ilium, pneumatised: absent (0); present (1) (Wilson & Upchurch, 2009; Mannion *et al*., 2013).

C250. Pubis, obturator foramen, in lateral view is: subcircular (0); oval or elliptical, with long axis oriented in same plane as long axis of pubis (1) (Mannion & Calvo, 2011; Mannion *et al*., 2013).

C251. Pubis, anterior margin of distal end strongly concave in lateral view, such that the distal end forms a prominent, anteriorly expanded boot: absent (0); present (1) (Naish & Martill, 2001; Mannion *et al*., 2013).

C252. Ischium, acetabular margin, in lateral view: flat or mildly concave (0); strongly concave, such that the pubic articular surface forms an anterodorsal projection (1) (D’Emic, 2012; Mannion *et al*., 2013).

C253. Ischium, symphysis between the ischia: terminates at the base of the proximal plates (emarginate distal to pubic articulation) (0); extends along the ventral edges of the proximal plates as well as the distal shafts, so that there is no V-shaped gap between the anterior ends of the ischia in dorsal view (no emargination distal to pubic articulation) (1) (McIntosh, 1990; Upchurch, 1998; Wilson, 2002).

C254. Ischium, long-axis of shaft, if projected upwards: passes through the lower part of the acetabular margin or the upper part of the pubic articular surface (i.e. it is approximately 60° to the horizontal in lateral view) (0); passes through the upper part of the acetabular margin or even approaches the rim of the iliac articulation (i.e. the shaft is at approximately 80° to the horizontal) (1) (Upchurch, 1995, 1998).

C255. Femur, proximolateral margin, above the lateral bulge: level with or lateral to the lateral margin of the distal half of the shaft (0); medial to the lateral margin of the distal half of the shaft (1) (McIntosh, 1990; Calvo & Salgado, 1995; Salgado *et al*., 1997; Royo-Torres, 2009; Royo-Torres *et al*., 2012; Mannion *et al*., 2013).

C256. Femur, anteroposterior thickness of lateral margin of proximal third: relatively constant with main body of femur (0); narrows to form a flange-like trochanteric shelf, forming a medially bounding vertical ridge along the posterior surface (1) (Mannion *et al*., 2013).

C257. Femur, proximodistally elongate midline ridge (linea intermuscularis cranialis) on anterior face, extending along most of shaft length: absent (0); present (1) (Otero, 2010; D’Emic, 2012).

C258. Femur, fourth trochanter: not visible in anterior view (0); visible in anterior view (1) (Gallina & Apesteguía, 2005; Whitlock, 2011).

C259. Femoral distal condyles, orientation relative to long axis of femoral shaft: bevelled dorsolaterally approximately 10° (tibial condyle extends further distally than fibular condyle) (0); perpendicular (tibial and fibular condyles extend approximately the same distance distally) (1); bevelled dorsomedially approximately 10° (fibular condyle extends further distally than tibial condyle) (2) (Wilson, 2002; Mannion *et al*., 2013) [ordered].

C260. Tibia, cnemial crest projects: laterally (0); anteriorly or anterolaterally (1) (Wilson & Sereno, 1998; Mannion *et al*., 2013; note that the tibia is oriented so that the flat, mediolaterally wide triangular surface of the distal end faces anteriorly).

C261. Tibia, lateral edge of proximal end forms a pinched out projection, posterior to cnemial crest (the “second cnemial crest” of Bonaparte *et al*., 2000): present (0); absent (1) (Bonaparte *et al*., 2000; Mannion *et al*., 2013).

C262. Fibula, proximal end with anteromedialy directed crest extending into a notch behind the cnemial crest of the tibia: absent (0); present (1) (Wilson & Upchurch, 2009; D’Emic, 2012; Mannion *et al*., 2013).

C263. Fibula, lateral muscle scar is: oval in outline (0); formed from two vertically elongate, parallel ridges (1) (Powell, 1992; Upchurch, 1998).

C264. Fibula, shaft in lateral view: straight (0); sigmoidal (1) (Canudo *et al*., 2008; Royo-Torres, 2009).

C265. Astragalus, in dorsal (or proximal) view: rectangular, with anteroposterior lengths of medial and lateral margins subequal (or medial margin greater) (0); wedge-shaped, narrowing anteroposteriorly towards its medial end, such that it has a reduced anteromedial corner (1) (Cooper, 1981; Upchurch, 1995, 1998; Wilson & Sereno, 1998).

C266. Astragalus, ascending process: does not extend to the posterior margin of the astragalus (usually limited to anterior two-thirds of astragalus) (0); extends to the posterior margin of the astragalus (1) (Wilson & Sereno, 1998; Wilson, 2002).

C267. Astragalus, laterally-directed ventral shelf underlies the distal end of the fibula: present (0); absent (1) (Wilson & Upchurch, 2009; Mannion *et al*., 2013).

C268. Astragalus: caps most, or all, of the distal end of the tibia (0); reduced so that medial edge of tibia is uncapped (1) (Wilson & Upchurch, 2009; Ksepka & Norell, 2010).

C269. Astragalus, posterior margin bears a tongue-like projection posteromedial to the ascending process, which is separated from the latter by a groove: present (0); absent (1) (D’Emic, 2012; Mannion *et al*., 2013).

C270. Calcaneum: present (0); absent (1) (McIntosh, 1990; Upchurch, 1995, 1998).

C271. Calcaneum, shape in proximal view: subcircular (0); subrectangular (1) (Wilson & Upchurch, 2009; Mannion *et al*., 2013).

C272. Metatarsals, metatarsal I with a prominent ventrolateral expansion along its distal half, such that the distal end expands further laterally than the proximal end: absent (0); present (1) (Berman & McIntosh, 1978; McIntosh, 1990; Upchurch, 1995, 1998; D’Emic *et al*., 2011; Mannion *et al*., 2013).

C273. Metatarsals, lateral margin of metatarsal II in proximal view: concave (0); straight (1) (Mannion *et al*., 2013).

C274. Metatarsals, metatarsal II distal articular surface extends up on to the dorsal surface (extending proximally approximately 25% of metatarsal length and most prominently along medial half): absent (0); present (1) (D’Emic *et al*., 2011; Mannion *et al*., 2013).

C275. Metatarsals, medial surface of the proximal portion of metatarsal IV concave (for reception of metatarsal III): absent (0); present (1) (D’Emic *et al*., 2011; D’Emic, 2012).

C276. Metatarsals, distal end orientation of metatarsal IV: perpendicular to long axis of bone (0); bevelled to face medially (1) (D’Emic, 2012).

C277. Pedal digit IV: has at least three phalanges (0); has two phalanges or fewer (1) (Upchurch, 1995, 1998; Upchurch *et al*., 2004).

C278. Pedal unguals, tuberosity on the ventral margin, along distal half: absent (0); present (1) (Canudo *et al*., 2008; Mannion *et al*., 2013).

C279. Osteoderms: absent (0); present (1) (Wilson, 2002; note that taxa are only coded as the plesiomorphic state when osteoderms are not found associated with a relatively complete postcranial skeleton or a specimen preserving numerous axial elements).

C280. Humerus, strong bulge or tuberosity (site for M. scapulohumeralis anterior) on the lateral margin of the posterior surface (usually visible in anterior view), approximately level with the most prominently developed portion of the deltopectoral crest: absent or weakly developed, and not visible in anterior view (0); present, forms a distinct lateral bulge that interrupts the line of the lateral humeral margin in anterior view (1) (Borsuk-Białynicka, 1977; Upchurch *et al*., 2015).

C281. Ulna, posterior process of proximal end: weakly developed, so that the proximal profile of the ulna is ‘V’-shaped (formed by the anteromedial and anterolateral processes) (0); strongly developed, so that the proximal profile of the ulna is ‘T’- or ‘Y’-shaped, and there is a deep fossa between the anteromedial and posterior processes, rivalling the radial fossa in depth (1) (Upchurch *et al*., 2015).

C282. Ulna, shape of the distal end: comma-shaped, with tapering curved anterior process associated with an anteromedial fossa for reception of the radius (0); elliptical or oval in outline, with the anteromedial fossa strongly reduced or absent (1) (Upchurch *et al*., 2015).

C283. Radius, profile of proximal end: ‘D’-shaped or elliptical (0); oval or subtriangular, with marked tapering towards the medial process (1) (Upchurch *et al*., 2015).

C284. Radius, ridge or flange on medial margin, near proximal end, for attachment of the M. biceps brachii and M. brachialis inferior: absent or very weakly developed (0); present, projecting beyond the medial margin of the main radial shaft (1) (Borsuk-Białynicka, 1977; Upchurch *et al*., 2015).

C285. Radius, posterior margin of distal end: lacks condylar-like processes and fossa (0); forms two low rounded processes (posteromedial and posterolateral), with a shallow fossa between them (1) (D'Emic, 2013; Upchurch *et al*., 2015).

C286. Snout, shape in dorsal view, Premaxillary-Maxillary Index (PMI): 60% or less (0); >60% to 75% (1); greater than 75% (2) (Upchurch, 1998; Whitlock, 2011; Poropat *et al*., 2016; note that this can be calculated using the lower jaw when the skull is incomplete) [ordered].

C287. Premaxilla, shape of ascending process in lateral view: convex (0); concave, with a large dorsal projection (1); sub-rectilinear and directed posterodorsally (2) (Whitlock, 2011).

C288. Maxilla, anterior (dentigerous) portion of lateral surface excavated by dorsoventrally elongate, deep vascular grooves: absent (0); present (1) (Wilson, 2002, 2005).

C289. Maxilla, foramen anterior to the preantorbital fenestra: absent (0); present (1) (Zaher *et al*., 2011).

C290. Maxilla, position of external opening of the preantorbital fenestra: lies below antorbital fenestra (0); lies anterior to antorbital fenstra, with the posterior margin of the preantorbital fenestra lying entirely anterior to the anterior margin of the antorbital fenestra (1) (Marpmann *et al*., 2015; Poropat *et al*., 2016).

C291. Maxilla, preantorbital fenestra development: weakly developed, shallow fossa (difficult to distinguish from posterior maxillary foramen) (0); deep, sharp-lipped fossa (1) (Zaher *et al*., 2011; Carballido *et al*., 2012; Poropat *et al*., 2016).

C292. Maxilla, posterior extent of dorsal (ascending) process: anterior to, or level with, posterior end of main body (0); extending posterior to posterior end of main body (1) (Whitlock, 2011).

C293. Maxilla, ventral margin of jugal process: reduced in dorsoventral height, such that the ventral margin is strongly emarginated relative to the remainder of the ventral margin of the maxilla (0); continuous with ventral margin of remainder of maxilla, or very gently emarginated (1) (Curry Rogers, 2005; Poropat *et al*., 2016).

C294. Maxilla, contact with quadratojugal: absent or small (i.e. no more than a point contact) (0); extensive (1) (Upchurch, 1995; Poropat *et al*., 2016).

C295. Jugal, dorsal process: present (0); absent (1) (Tschopp *et al*., 2015).

C296. Jugal, contact with ectopterygoid: present (0); absent, ectopterygoid contacts maxilla instead (1) (Upchurch, 1995).

C297. Frontal, anteroposterior length to transverse width ratio: 1.0 or greater (0); less than 1.0 (1) (Whitlock, 2011; Tschopp & Mateus, 2013; Poropat *et al*., 2016).

C298. Frontal, lateral margin: expands posteriorly, orbital margin concave in dorsal view (0); unexpanded posteriorly, orbital margin straight or convex in dorsal view (1) (Whitlock *et al*., 2010; Poropat *et al*., 2016).

C299. Frontal, contribution to margin of supratemporal fenestra: present (0); absent, frontal excluded from anterior margin of fenestra by a postorbital-parietal contact (1) (Wilson & Sereno, 1998).

C300. Orbit, anterior-most point: anterior to the anterior extremity of lateral temporal fenestra (0); approximately level with or posterior to anterior extent of lateral temporal fenestra (1) (Gauthier, 1986; Upchurch, 1995; Poropat *et al*., 2016).

C301. Lateral temporal fenestra, shape in lateral view: taller than wide anteroposteriorly and subtriangular (anteroposteriorly broader ventral margin and narrower dorsal apex) (0); linear, slit-like, crescentic (longer anteroposteriorly than high dorsoventrally) (1) (Harris, 2006; Poropat *et al*., 2016).

C302. Postorbital, posterior (squamosal) process: present as a distinct process (0); absent (1) (Wilson, 2002, 2005a).

C303. Parietal, relative height of suture with frontal: lies level with or above the dorsal surfaces of the frontals and parietals (0); lies below the dorsal surfaces of the parietals and frontals (i.e. in a deep transverse trough) (1) (Curry Rogers, 2005; Poropat *et al*., 2016).

C304. Parietal, elevation of anterior margin creates a step-like curving crest transversely, where the parietal meets the frontal: absent (0); present (1) (Curry Rogers & Forster, 2004; Curry Rogers, 2005; Poropat *et al*., 2016).

C305. Pterygoid, morphology: robust element (0); plate-like, with its three processes coplanar (1) (Curry Rogers & Forster, 2004; Wilson, 2005a; Poropat *et al*., 2016).

C306. Pterygoid, sutural contact with ectopterygoid: anteroposteriorly elongate, along the medial or lateral surface (0); anteroposteriorly short, restricted to the anterior tip of the pterygoid (1) (Zaher *et al*., 2011; Poropat *et al*., 2016).

C307. Pterygoid, palatobasal contact for basipterygoid articulation with a convex, rocker-like articular surface: absent (0); present (1) (Wilson, 2002).

C308. Supraoccipital, longitudinal groove along posterodorsal surface: absent (0); present (i.e. sagittal crest divided into two subparallel parasagittal crests with central groove) (1) (Curry Rogers & Forster, 2004; Curry Rogers, 2005; González Riga *et al*., 2009).

C309. Supraoccipital-exoccipital-opisthotic, paired facets for articulation with the proatlas: absent (0); present (1) (Poropat *et al*., 2016).

C310. Parasphenoid rostrum, cross-sectional shape: triangular (0); transversely thin, sheet-like (1) (Berman & McIntosh, 1978; Upchurch, 1995, 1998; Tschopp *et al*., 2015; Poropat *et al*., 2016)

C311. Basipterygoid processes, shape: widely diverging at 30° or more (0); narrowly diverging at less than 30° (1) (Upchurch, 1995, 1998; Mannion *et al*., 2012).

C312. Basipterygoid processes, orientation in lateral view: directed 80° or more to skull roof (normally perpendicular) (0); angled less than 80° to skull roof (anteroventrally directed) (1) (McIntosh, 1990; Wilson, 2002; Whitlock, 2011; Mannion *et al*., 2012; Poropat *et al*., 2016).

C313. Basioccipital, orientation of occipital condyle relative to the horizontal plane (in lateral view with supraoccipital held in a vertical plane): 60° or less (0); greater than 60° (typically close to 90°) (1) (Upchurch, 1995, 1998).

C314. Basal tubera, angle of divergence in posterior view: less than 50° (0); more than or equal to 50° (1) (Curry Rogers, 2005; Poropat *et al*., 2016).

C315. Basisphenoid, relative position of the external opening for cranial nerve VI: lies ventral, and generally close, to the opening for cranial nerve III (0); lies anteroventral to, and more distant from, the opening for cranial nerve III (1) (Remes, 2009; Poropat *et al*., 2016).

C316. Basisphenoid, opening for cranial nerve VI: penetrates the pituitary fossa (0); does not penetrate the pituitary fossa (1) (Paulina Carabajal, 2012; Poropat *et al*., 2016).

C317. Basioccipital+exoccipital-opisthotic, number of exits for cranial nerve XII: 2 (0); 1 (1) (Paulina Carabajal, 2012; Poropat *et al*., 2016).

C318. Basisphenoid, position of the external foramen of the internal carotid artery: lateral to basipterygoid process (0); medial to basipterygoid process (1) (Paulina Carabajal, 2012; Poropat *et al*., 2016).

C319. Dentary, angle between the long-axis of the anterior margin (mandibular symphysis) and the long-axis of the main body of the dentary, in lateral view: greater than 90°, with the dorsal margin of the dentary extending further anteriorly than the ventral margin (0); approximately 90°, with the dorsal and ventral margins extending an equal distance anteriorly (1) (Salgado & Calvo, 1997; Upchurch, 1998; Wilson, 2002; Poropat *et al*., 2016).

C320. Tooth crowns, longitudinal groove paralleling mesial and distal margins on the labial surface: labial grooves present (0); absent (1) (Upchurch, 1995; Poropat *et al*., 2016).

C321. Atlantal intercentrum, ventral margin of posterior surface: straight or convex (0); concave, forming ventrolateral projections (1) (González Riga & Ortíz David, 2014; Poropat *et al*., 2016).

C322. Axis, aEI (average elongation index: anteroposterior length of centrum (excluding articular ball if present) divided by mean average value of the mediolateral width and dorsoventral height of posterior articular surface of centrum): 2.0 or greater (0); less than 2.0 (1) (Upchurch *et al*., 2007; Poropat *et al*., 2016).

C323. Postaxial cervical centra, pneumatization of lateral surface: lateral pneumatic opening occupies approximately anterior two-thirds of centrum or more (0); reduced and restricted to less than the anterior two-thirds of the centrum (1) (Whitlock, 2011; Poropat *et al*., 2016).

C324. Postaxial cervical centra, midline notch on the dorsal margin of the posterior articular surface: absent (0); present (1) (Carballido *et al*., 2012).

C325. Postaxial anterior cervical vertebrae, prezygapophyses: extend anterior to the anterior tip of the condyle (0); terminate level with or posterior to the anterior tip of the condyle (1) (Curry Rogers, 2005, 2009; Poropat *et al*., 2016).

C326. Postaxial anterior cervical neural spines, orientation of posterior margin in lateral view: dorsal (vertical) or anterodorsal (0); posterodorsal (1) (Curry Rogers, 2005).

C327. Middle cervical neural spines, height to arch height ratio: 2.0 or lower (0); greater than 2.0 (1) (Rauhut *et al*., 2005; Whitlock, 2011).

C328. Posterior (usually just the posterior-most) cervical neural arches, postzygapophyses: terminate at or beyond the posterior edge of the centrum (0); terminate in front of the posterior edge of the centrum (1) (Tschopp & Mateus, 2013; Poropat *et al*., 2016).

C329. Posterior cervical neural spines, horizontal, rugose ridge immediately below spine summit on lateral surface: absent, spinodiapophyseal fossa fades out gradually dorsally (0); present, serves as distinct dorsal edge of the spinodiapophyseal fossa (1) (Tschopp & Mateus, 2013).

C330. Posteriormost cervical and anteriormost dorsal neural spines, shape in anterior/posterior view: taper dorsally, or mediolateral width remains constant along length (0); expand dorsally, with a strongly convex dorsal margin (‘paddle-shaped’) (1) (González Riga, 2005; D'Emic, 2012; Poropat *et al*., 2016).

C331. Cervical ribs, dorsal surface of proximal portion of shaft: excavated, forming a longitudinal groove (0); unexcavated (1) (Poropat *et al*., 2015a, 2016).

C332. Anterior dorsal centra, ventral keel on midline: absent (0); present (1) (Mannion *et al*., 2012; Poropat *et al*., 2016).

C333. Middle–posterior dorsal centra, ventral surface: flat or transversely convex (0); transversely concave, between ventrolateral ridges (1) (Upchurch *et al*., 2004; Curry Rogers, 2005; Poropat *et al*., 2016).

C334. Middle–posterior dorsal centra, lateral pneumatic foramina divided by internal ridge/s: absent (0); present (1) (Salgado *et al*., 1997; Mannion *et al*., 2012).

C335. Anterior dorsal neural arches, shape of anterior neural canal opening: height greater than or equal to width (0); height is less than width (1) (Curry Rogers, 2005; Poropat *et al*., 2016).

C336. Anterior–middle dorsal neural arches, vertical midline ridge (‘median infrapostzygapophyseal lamina’) extending from roof of neural canal to ventral midpoint of postzygapophyses/intrapostzygapophyseal lamina (TPOL): absent (0); present (1) (González Riga, 2003; Curry Rogers, 2005, 2009; Gallina, 2011; Gallina & Apesteguía, 2011).

C337. Anterior–middle dorsal neural arches, zygapophyseal articulation angle: between horizontal and less than 40° to the horizontal (0); strongly dorsomedially oriented (40° or more) (1) (Carballido *et al*., 2012; Poropat *et al*., 2016).

C338. Middle–posterior dorsal neural arches, neural canal in anterior view: entirely surrounded by the neural arch (0); enclosed in a deep fossa in the dorsal surface of the centrum (i.e. much of the canal is enclosed laterally by pedicels that are part of the centrum rather than the neural arch) (1) (Carballido *et al*., 2012; Poropat *et al*., 2016).

C339. Middle–posterior dorsal neural arches, position of parapophysis: posterior to the vertical plane defined by the anterior margin of the centrum (excluding any convex articular condyle) (0); level with, or anterior to, the vertical plane defined by the margin of the centrum (excluding any convex articular condyle) (1) (Tschopp & Mateus, 2013).

C340. Middle–posterior dorsal neural arches, anterior centradiapophyseal lamina (ACDL): absent (0); present (1) (Mannion *et al*., 2012; Poropat *et al*., 2016).

C341. Posterior dorsal neural arches, zygapophyseal articulation angle relative to horizontal line: less than 30°, usually close to horizontal (0); steeply oriented, 30° or greater (1) (Carballido *et al*., 2012; Poropat *et al*., 2016).

C342. Middle–posterior dorsal neural spines (single, not bifid), SPRLs: remain separate or converge at about spine midheight (or above) to form a dorsally restricted median composite lamina (SPRF well-developed and occupies the ventral half of the anterior spine surface) (0); SPRLs, if present, are short and merge into the PRSL close to the base of the spine (the PRSL may extend between the bases of the SPRLs to the top of the TPRL) (1) (Upchurch, 1995; Whitlock, 2011; Carballido *et al*., 2012; Poropat *et al*., 2016).

C343. Middle–posterior dorsal neural spines, postspinal lamina: does not extend ventral to the neural spine (0); extends ventral to the neural spine, beyond the postzygapophyseal articular surfaces (1) (Poropat *et al*., 2016; note that only taxa scored as “0” for C169 are scored for this character).

C344. Middle–posterior dorsal neural spines, anterior spinodiapophyseal lamina (aSPDL): absent (0); present (1) (Upchurch *et al*., 2004; Poropat *et al*., 2016; note that the presence of an aSPDL can only be confirmed when a pSPDL is also present).

C345. Middle–posterior dorsal neural spines, SPDL bifurcates at its dorsal end to create a SPDL-F: absent (0); present (1) (Poropat *et al*., 2016; note that this refers to a bifurcation of the posterior SPDL when there are two SPDLs, rather than just the presence of an aSPDL and pSPDL).

C346. Sacrum, ratio of mediolateral width across sacral vertebrae and ribs (taken at midlength on the coossified sacrum) to average length of a sacral centrum: less than 4.0 (0); 4.0 or higher (1) (Upchurch, 1998).

C347. Sacral centra, ratio of mediolateral width of middle sacral centra to first and last sacral centra: approximately constant, ratio less than 1.3 (0); 1.3 or greater (1) (Salgado *et al*., 2005; D'Emic & Wilson, 2011; Poropat *et al*., 2016).

C348. Sacral neural spines, all fused, forming a dorsal ‘platform’: absent (0); present (1) (Martínez *et al*., 2004; Poropat *et al*., 2016).

C349. First caudal centrum, anterior articular face shape: flat or concave (0); convex (1) (Wilson, 2002; Whitlock, 2011; Poropat *et al*., 2016).

C350. Anterior–middle caudal centra (excluding Cd1), comparison of anterior and posterior articular faces: anterior face more concave than posterior one, or these two faces are equally concave (0); posterior face more deeply concave than anterior face (1) (González Riga *et al*., 2009; Carballido *et al*., 2012; D'Emic *et al*., 2013; Poropat *et al*., 2016).

C351. Middle caudal centra with convex posterior articular surface, condyle dorsally displaced: absent (0); present (1) (new character: based on González Riga, 2003).

C352. Anteriormost caudal neural arches, prezygapophyses curve downwards (‘droop’) at their distal ends: absent (0); present (1) (Santucci & Arruda-Campos, 2011).

C353. Anterior caudal neural spines, anterior expansion of lower portion of spinoprezygapophyseal lamina (SPRL): absent (0); present (‘SPRL-process’) (1) (D'Emic, 2012; Mannion *et al*., 2012; note that this is best observed in lateral view).

C354. First caudal rib, subtriangular process projects posteriorly at approximately midlength: absent (0); present (1) (Mannion & Calvo, 2011; Poropat *et al*., 2016).

C355. Anteriormost caudal ribs, tubercle on dorsal surface at approximately midlength: absent (0); present (1) (Kellner *et al*., 2005; D'Emic *et al*., 2013; Poropat *et al*., 2016).

C356. Anterior–middle chevrons, articular facet surface: flat or anteroposteriorly convex (0); divided into anterior and posterior facets by a furrow (1) (Powell, 1987, 2003; Santucci & Arruda-Campos, 2011; D'Emic, 2012).

C357. Anterior–middle chevrons, posteroventrally directed ridge or bulge on lateral surface of distal half of proximal ramus: absent (0); present (1) (Santucci & Arruda-Campos, 2011; Poropat *et al*., 2016).

C358. Scapula, ventrolateral margin of acromion, anteroposteriorly concave region posterior to glenoid, followed by a flattened area: absent (0); present (1) (Poropat *et al*., 2015a, 2016).

C359. Scapular blade, ridge on medial surface, close to junction with acromial plate and near dorsal margin: absent (0); present (1) (Sanz *et al*., 1999; Upchurch *et al*., 2004).

C360. Scapular blade, orientation of blade long-axis with respect to coracoid articulation: more than 70° (usually approximately perpendicular) (0); 70° or less (1) (Wilson, 2002; Poropat *et al*., 2016).

C361. Coracoid, glenoid: does not expand strongly laterally relative to the lateral surface of the coracoid (0); expands prominently laterally and curves dorsolaterally so that part of the glenoid articular surface can be seen in lateral view (1) (Poropat *et al*., 2016).

C362. Sternal plate, shape of posterior margin in dorsal/ventral view: convex (0); straight (1) (González Riga, 2003; González Riga *et al*., 2009).

C363. Sternal plate, anteroposteriorly directed ridge on ventral surface, at the anterior end: absent (0); present (1) (Sanz *et al*., 1999; Upchurch *et al*., 2004).

C364. Humerus, proximal margin in anterior/posterior view: straight or convex (0); sinuous, as a result of a prominently developed process (attachment site for M. supracoracoideus) on the lateral margin of the proximal end (1) (Upchurch, 1998; González Riga, 2003; González Riga *et al*., 2009; Poropat *et al*., 2016).

C365. Humerus, proximal end: expands laterally relative to the shaft, giving the humerus an hourglass outline in anterior view (0); asymmetrical, with no expansion of lateral margin relative to shaft (1) (Tschopp *et al*., 2015; Poropat *et al*., 2016).

C366. Humerus, humeral head forms a prominent subcircular process on the posterior surface of the proximal end: absent (0); present (1) (Bonaparte *et al*., 2006; Upchurch *et al*., 2015; Poropat *et al*., 2016).

C367. Humerus, prominent vertical ridge extends along the lateral margin of the posterior surface, from the proximolateral corner to approximately the level of the deltopectoral crest (this ridge defines the lateral margin of the lateral triceps fossa and causes this fossa to be much deeper than the medial one): absent (0); present (1) (Poropat *et al*., 2016).

C368. Humerus, tuberosity for attachment of the M. coracobrachialis on the anterior surface of the proximal third: absent (0); present (1) (Powell, 2003; Harris, 2007; Otero, 2010; Poropat *et al*., 2016).

C369. Humerus, deltopectoral crest, mediolateral thickness of anterior attachment surface: approximately constant along length (0); distal half mediolaterally expanded relative to proximal half (often doubling in thickness) (1) (Wilson, 2002; Poropat *et al*., 2016; note that the anterior attachment surface of taxa with a medially deflected deltopectoral crest faces primarily medially).

C370. Humerus, ratio of maximum mediolateral width of distal end to proximodistal length: 0.30 or greater (0); less than 0.30 (1) (Poropat *et al*., 2016).

C371. Radius, beveling of distal end relative to long-axis of shaft: restricted to lateral half (0); extends across the entire distal end (1) (Poropat *et al*., 2016).

C372. Ulna, angle between long-axes of anteromedial and anterolateral processes in proximal end view: 80° or greater (usually approximately a right-angle) (0); less than 80° (acute) (1) (Tschopp *et al*., 2015).

C373. Metacarpals, longest metacarpal to radius proximodistal length ratio: less than 0.50 (0); 0.50 or greater (1) (Poropat *et al*., 2016).

C374. Metacarpals, metacarpal II, ratio of minimum transverse width of shaft to metacarpal length: 0.2 or higher (0); less than 0.2 (1) (Sekiya, 2011).

C375. Metacarpals, metacarpal III: longest metacarpal (0); shorter than at least one other metacarpal (1) (Curry Rogers, 2005; Poropat *et al*., 2016).

C376. Metacarpals, metacarpal IV, distal end profile: subrectangular (0); possesses small pointed lateral and medial projections such that the dorsal margin is longer than the ventral margin, producing a dorsoventrally compressed hexagonal or trapezoidal outline (1) (Poropat *et al*., 2016).

C377. Metacarpals, metacarpal V, ratio of proximal end long axis diameter to that of metacarpal I: less than 1.0 (0); 1.0 or greater (1) (D'Emic, 2012; Poropat *et al*., 2016).

C378. Metacarpals, metacarpal V, ratio of proximal end long axis diameter to that of metacarpal IV: equal or smaller (0); larger than that for metacarpal IV (1) (Poropat *et al*., 2015b, 2016).

C379. Metacarpals, metacarpal V with a medially biased flange-like swelling along proximal half of ventral surface: absent (0); present (1) (Apesteguía, 2005a; Mannion & Calvo, 2011; Poropat *et al*., 2016).

C380. Ilium, ratio of dorsoventral height of iliac blade above pubic peduncle to anteroposterior length of ilium: less than 0.35 (0); 0.35 or greater (1) (Tschopp *et al*., 2015; Poropat *et al*., 2016).

C381. Ilium, projected line (chord) connecting articular surfaces of ischiadic and pubic processes: passes ventral to ventral margin of postacetabular portion of ilium (0); passes through or dorsal to ventral edge of postacetabular portion of ilium (1) (Upchurch, 1998).

C382. Ilium, orientation of the pubic peduncle with respect to the long axis of the ilium: anteriorly deflected (0); perpendicular (1) (Salgado *et al*., 1997).

C383. Ilium, protuberance on the lateral surface of the ischiadic articulation: absent (0); present (1) (Borsuk-Białynicka, 1977; Poropat *et al*., 2015a, 2016).

C384. Pubis, proximodistally oriented ridge on lateral surface of blade, separated from the anterior margin of the pubis by a longitudinal groove: absent (0) present (1) (Powell, 2003; Salgado and Carvalho, 2008; Otero, 2010; Poropat *et al*., 2016).

C385. Pubis, distal end transversely expanded along lateral surface relative to shaft: present (0); absent (laminar distal blade) (1) (Curry Rogers, 2005; Poropat *et al*., 2016).

C386. Ischium, iliac articular surface, anteroposterior length to mediolateral width ratio: 1.0 or greater (0); less than 1.0 (1) (Poropat *et al*., 2016).

C387. Ischium, ridge (for attachment of M. flexor tibialis internus III) on lateral surface of the lower part of the proximal plate/proximal portion of shaft, close to the posterior/dorsal margin of ischium: associated with parallel groove, posterior/dorsal to ridge (0); groove absent (1) (Sereno *et al*., 2007; D'Emic, 2012; Poropat *et al*., 2016).

C388. Femur, femoral head, projection: directed medially (0); directed dorsomedially (1) (Upchurch *et al*., 2004; Curry Rogers, 2005; Poropat *et al*., 2016).

C389. Femur, ratio of mediolateral breadth of tibial condyle to breadth of fibular condyle: greater than 0.8 (0); 0.8 or less (1) (Wilson, 2002; Poropat *et al*., 2016).

C390. Femur, shape of distal condyles: articular surface restricted to distal portion of femur (0); expanded onto anterior portion of femoral shaft (1) (Wilson & Carrano, 1999; Wilson, 2002).

C391. Tibia to femur length ratio: less than 0.6 (0); 0.6 or greater (1) (Gauthier, 1986; Poropat *et al*., 2016).

C392. Fibula, articular surface of lateral trochanter: not visible in anterior view (0); visible in anterior view (1) (Poropat *et al*., 2016).

C393. Fibula, distal end mediolateral width to anteroposterior width ratio: 0.8 or less (0); greater than 0.8 (1) (Poropat *et al*., 2016).

C394. Fibula, distal end profile: elliptical or semicircular (with a straight medial margin) (0); subtriangular (with a rounded or sharper apex projecting laterally or anterolaterally where flattened anterolateral and posterolateral margins meet) (1) (Poropat *et al*., 2016).

C395. Metatarsals, metatarsal V, proximal end: dorsoventrally expanded relative to shaft, with a domed dorsal margin (0); not expanded relative to shaft (1) (Poropat *et al*., 2016).

C396. Metatarsals, metatarsal V, tubercle or ridge on ventral surface, at approximately midlength, equidistant from the medial and lateral margins: absent (0); present (1) (Poropat *et al*., 2016).

C397. Pedal digit I, proximal articular surface of ungual (phalanx I-2): perpendicular to long axis of ungual (0); bevelled so that the proximal articular surface faces proximolaterally and thus lies at a distinct angle to the long axis of the ungual (1) (Wilson & Upchurch, 2009; Poropat *et al*., 2016).

C398. Basioccipital, fossa on lateral surface, extending from base of occipital condyle to base of basal tubera: absent (0); present (1) (Tschopp, Mateus & Benson, 2015).

C399. Basicranium, cranial nerve opening II (optic foramen): single opening (0); medially divided to form two foramina (1) (Sander *et al*., 2006).

C400. Surangular, anterior foramen: absent (0); present (1) (Tschopp *et al*., 2015).

C401. Splenial, position of anterior end relative to mandibular symphysis: posterior to symphysis (0); participates in symphysis (1) (Upchurch, 1998).

C402. Teeth, D-shaped crown morphology in labial/lingual view: narrows mesiodistally along its apical third (0); narrows mesiodistally along its apical half, giving it a ‘heart’-shaped outline (1) (new character: based on Royo-Torres, Cobos & Alcalá, 2006; Mateus, Mannion & Upchurch, 2014).

C403. Middle–posterior cervical neural arches, vertical midline lamina (part of the interprezygapophyseal lamina [TPRL]) divides the centroprezygapophyseal fossa (CPRF) into two fossae: absent (0); present (1) (Upchurch & Martin, 2002; Curry Rogers, 2009).

C404. Middle–posterior cervical neural arches, vertical midline lamina (part of the interpostzygapophyseal lamina [TPOL]) divides the centropostzygapophyseal fossa (CPOF) into two fossae: absent (0); present (1) (Upchurch & Martin, 2002; Curry Rogers, 2009).

C405. Middle cervical neural spines, lateral fossa at the base of the prezygapophyseal process bounded by SPRL, PRDL and PODL: absent (0); present (1) (Harris, 2006; Tschopp & Mateus, 2013).

C406. Middle and posterior cervical neural spines, lateral surface between PRDL, PODL, SPOL (i.e. the spinodiapophyseal fossa [SDF]): has 3 or more coels separated from each other by low ridges: absent (0) present (1) (new character based on Mannion & P. Upchurch, unpublished data).

C407. Cervical ribs, anterior projection extends beyond anterior margin of centrum (including condyle): present (0); absent (1) (new character).

C408. Sacral ribs, Sv2 ribs: emanate solely from Sv2 (0); emanate from Sv2, with a contribution from Sv1 (1) (new character).

C409. Anteriormost caudal centra, ACDL: absent, or represented by no more than a faint ridge (0); present, well defined or sheet-like (1) (new character based on Wilson, 2002; Mannion & P. Upchurch, unpublished data).

C410. Anterior–middle caudal neural arches: spinopostzygapophyseal lamina (SPOL) shape: SPOL grades smoothly toward postzygapophyses (0); SPOL abruptly ends near the anterior margin of the postzygapophyseal facet, and postzygapophyses sharply set off from neural spine, often projecting as distinct processes (1) (D’Emic *et al*., 2016; note that this feature is usually present in the last few anterior and first few middle caudal vertebrae).

C411. Anterior–middle caudal neural arches, anteroposteriorly oriented ridge and fossa (‘shoulder’) between prezygapophyses and postzygapophyses: absent (0); present (1) (D’Emic *et al*., 2016; note that this feature is usually present in the last few anterior and first few middle caudal vertebrae).

C412. Radius, proximal to distal end anteroposterior length ratio: 0.5 or greater (0); less than 0.5 (1) (new character: based on Mateus, Mannion & Upchurch, 2014).

C413. Ulna, vertical groove and ridge structure on posterolateral surface of distal shaft: absent (0); present (1) (Royo-Torres, Cobos & Alcalá, 2006).

C414. Carpal bones, distal carpal mediolateral width to anteroposterior length ratio: less than 1.4 (0); 1.4 or greater (1) (new character: based on Royo-Torres *et al*., 2014; note that this is the largest carpal element in those taxa with more than one carpal).

C415. Metacarpal III, maximum mediolateral width to dorsoventral height of the proximal end ratio: less than 1.3 (0); 1.3 or greater (1) (new character).

C416. Tibia, tubercle (‘tuberculum fibularis’) on posterior (internal) face of cnemial crest: absent (0); present (1) (Harris, 2007; Tschopp *et al*., 2015).

**CHANGES IN CHARACTER SCORES**

Taxon scores for C1–397 follow those in the data matrix of Poropat *et al*. (2016), with the following changes (the first number denotes the character, and the number/symbol in parentheses denotes the new score):

*Atlasaurus*: 40 (3), 53 (0), 54 (?), 55 (?), 72 (0), 74 (0), 97 (1), 101 (0), 272 (1), 278 (0), 288 (0), 302 (0), 310 (0), 376 (0), 379 (1), 395 (0), 397 (1)

*Brachiosaurus*: 40 (3)

*Cedarosaurus*: 40 (3)

*Europasaurus*: 2 (1), 4 (0), 12 (1), 85 (0), 115 (1), 140 (0), 141 (0), 166 (1), 170 (0), 174 (0), 348 (0)

*Giraffatitan*: 40 (3)

*Lusotitan*: 67 (1), 68 (1), 118 (?), 119 (?), 120 (?), 145 (?), 146 (?), 159 (?), 161 (?), 167 (?), 172 (0), 173 (1), 208 (0/1), 235 (?), 249 (?), 372 (1), 387 (1)

*Sonorasaurus*: 22 (1), 28 (1), 29 (0), 34 (?), 42 (1), 43 (1), 44 (1), 46 (0), 47 (1), 48 (1), 50 (0), 53 (0), 57 (1), 65 (?), 69 (0), 151 (1), 152 (0), 183 (0), 192 (1), 217 (1), 224 (1), 225 (0), 226 (0), 227 (0), 228 (1), 229 (0), 230 (0), 231 (0), 232 (1), 233 (0), 236 (1), 244 (1), 245 (0), 249 (0), 262 (0), 263 (1), 273 (0), 274 (0), 280 (0), 281 (0), 282 (0), 283 (1), 285 (1), 369 (0), 370 (1), 371 (0), 376 (0), 392 (0), 393 (0), 394 (0), 396 (0)

*Tangvayosaurus*: 269 (1)

*Vouivria*: 17 (?), 18 (0), 26 (0), 36 (1), 44 (?), 45 (?), 46 (?), 47 (?), 49 (?), 51 (0), 64 (?), 67 (?), 70 (0), 115 (?), 131 (?), 142 (0), 147 (1), 151 (0), 173 (1), 176 (0), 178 (0), 180 (1), 188 (1), 189 (1), 190 (0), 194 (0), 212 (0), 213 (0), 215 (1), 216 (0), 217 (1), 218 (0), 219 (0), 220 (0), 224 (0), 226 (0), 228 (0), 231 (?), 236 (0), 243 (1), 244 (1), 245 (0), 246 (1), 249 (0), 261 (0), 262 (0), 263 (0), 264 (0), 269 (0); 272 (?), 274 (0), 281 (0), 282 (0), 333 (0), 346 (0), 353 (0), 358 (0), 359 (0), 361 (1), 367 (0), 369 (1), 371 (?), 376 (0), 382 (1), 387 (0), 388 (1), 389 (1), 391 (0), 394 (0)

**QUANTITATIVE VALUES FOR NEW CHARACTERS**

C414: *Shunosaurus*=1.19; *Omeisaurus*=1.30; *Mamenchisaurus*=1.48; *Camarasaurus*=1.14; *Apatosaurus*=1.43; *Diplodocus*=1.72; *Aragosaurus*=1.10; *Vouivria*=1.14; *Janenschia*=1.50; *Jobaria*=1.64; *Losillasaurus*=1.17; *Turiasaurus*=1.23 (note that other taxa can be scored for this character, but exact values are unknown).

C415: *Shunosaurus*=1.29; *Omeisaurus*=1.08; *Mamenchisaurus*=0.95; *Camarasaurus*=0.87; *Apatosaurus*=1.00; *Alamosaurus*=0.75; *Angolatitan*=1.08; *Atlasaurus*=1.43; *Chubutisaurus*=1.15; *Vouivria*=1.48; *Giraffatitan*=1.47; *Lapparentosaurus*=1.06; *Ligabuesaurus*=0.99; *Opisthocoelicaudia*=1.00; *Rapetosaurus*=0.98; *Saltasaurus*=1.04; *Venenosaurus*=1.33; *Epachthosaurus*=1.09; *Diamantinasaurus*=0.97; *Savannasaurus*=1.25; *Wintonotitan*=1.22; *Muyelensaurus*=1.09; *Jobaria*=1.86; *Zby*=1.07 (note that other taxa can be scored for this character, but exact values are unknown).

**REFERENCES**

**Apesteguía S. 2005a.** Evolution of the titanosaur metacarpus. In: Tidwell V, Carpenter K, eds. *Thunder-lizards: the sauropodomorph dinosaurs*. Bloomington; Indianapolis: Indiana University Press, 321–345.

**Apesteguía S. 2005b.** Evolution of the hyposphene-hypantrum complex within Sauropoda. In: Tidwell V, Carpenter K, eds. *Thunder-lizards: the sauropodomorph dinosaurs*. Bloomington, Indianapolis: Indiana University Press, 248–267.

**Barrett PM, Hasegawa Y, Manabe M, Isaji S, Matsuoka H. 2002.** Sauropod dinosaurs from the Lower Cretaceous of eastern Asia: taxonomic and biogeographical implications. *Palaeontology* **45:**1197–1217.

**Berman DS, McIntosh JS. 1978.** Skull and relationships of the Upper Jurassic Sauropod *Apatosaurus* (Reptilia, Saurischia). *Carnegie Museum Bulletin* **8:**1–35.

**Bolliger W, Burri P. 1967.** Versuch einer Zeitkorrelation zwischen Plattformcarbonaten und tiefermarinen Sedimenten mit Hilfe von Quarz-Feldspat-Schüttungen (mittlerer Malm des Schweizer Jura). *Eclogae Geologicae Helvetiae* **60:**491–507.

**Bonaparte JF. 1986.** The early radiation and phylogenetic relationships of the Jurassic sauropod dinosaurs, based on vertebral anatomy. In: Padian K, ed. *The beginning of the Age of Dinosaurs*. Cambridge: Cambridge University Press, 247–258.

**Bonaparte JF, Coria RA. 1993.** Un nuevo y gigantesco saurópodo titanosaurio de la Formación Río Limay (Albiano-Cenomaniano) de la Provincia del-Neuquen, Argentina. *Ameghiniana* **30:**271–282.

**Bonaparte JF, González Riga BJ, Apesteguía S. 2006.** *Ligabuesaurus leanzai* gen. et sp. nov. (Dinosauria, Sauropoda), a new titanosaur from the Lohan Cura Formation (Aptian, Lower Cretaceous) of Neuquén, Patagonia, Argentina. *Cretaceous Research* **27:**364–376.

**Bonaparte JF, Heinrich W-D, Wild R. 2000.** Review of *Janenschia* Wild, with the description of a new sauropod from the Tendaguru beds of Tanzania and a discussion on the systematic value of procoelous caudal vertebrae in the Sauropoda. *Palaeontographica, Abteilung A* **256:**25–76.

**Borsuk-Białynicka M. 1977.** A new camarasaurid sauropod *Opisthocoelicaudia skarzynskii* gen. n., sp. n. from the Upper Cretaceous of Mongolia. *Palaeontologica Polonica* **37:**5–63.

**Boullier A, Contini D, Pernin C. 1975.** L'Oxfordien des environs de Dole (Jura). *Annales Scientifiques de l’Université de Besançon. Géologie, 3e série* **23:**57–61.

**Broin F de, Barta-Calmus S, Beauvais L, Camoin G, Dejax J, Gayet M, Michard JG, Olivaux T, Roman J, Sigogneau-Russell D, Taquet P, Wenz S. 1992.** Réponse des auteurs. *Bulletin de la Société Géologique de France* **163:**523.

**Buffetaut E. 1988.** Les restes de dinosaures de l'Oxfordien supérieur de Damparis (Jura): preuves d'émersion sur place. *Revue de Paléobiologie* **7:**301–306.

**Buffetaut E. 1994.** The significance of dinosaur remains in marine sediments: an investigation based on the French record. *Berliner Geowissenschaftliche Abhandlungen. Reihe E, Paläobiologie* **13:**125–133.

**Calvo JO. 1994.** Jaw mechanics in sauropod dinosaurs. *Gaia* **10:**183–193.

**Calvo JO, Salgado L. 1995.** *Rebbachisaurus tessonei* sp. nov. a new Sauropoda from the Albian-Cenomanian of Argentina; new evidence on the origin of the Diplodocidae. *Gaia* **11:**13–33.

**Canudo JI, Royo-Torres R, Cuenca-Bescós G. 2008.** A new sauropod: *Tastavinsaurus sanzi* gen. et sp. nov. from the Early Cretaceous (Aptian) of Spain. *Journal of Vertebrate Paleontology* **28:**712–731.

**Carballido JL, Rauhut OWM, Pol D, Salgado L. 2011.** Osteology and phylogenetic relationships of *Tehuelchesaurus benitezii* (Dinosauria, Sauropoda) from the Upper Jurassic of Patagonia. *Zoological Journal of the Linnean Society* **163:**605–662.

**Carballido JL, Salgado L, Pol D, Canudo JI, Garrido A. 2012.** A new basal rebbachisaurid (Sauropoda, Diplodocoidea) from the Early Cretaceous of the Neuquén Basin; evolution and biogeography of the group. *Historical Biology* **24:**631–654.

**Cariou E. 2013.** La plateforme jurassienne au passage Oxfordien–Kimméridgien: dynamique sédimentaire et paléoenvironnements. Thèse de Doctorat, Université Claude Bernard–Lyon 1, Lyon, 411 pp.

**Cariou E, Olivier N, Pittet B, Mazin J-M, Hantzpergue P. 2014.** Dinosaur track record on a shallow carbonate-dominated ramp (Loulle section, Late Jurassic, French Jura). *Facies* **60:** 229–253.

**Chauve P, Kerrien Y, Pernin C, with collaboration of Cornet J, Féraud J. 1979.** Carte géologique de la France à 1/50 000 (n° 528), Dole (XXXII-24) Forêt de Chaux. Notice explicative. Bureau de Recherches Géologiques et Minières, Orléans, 32 pp.

**Chure D, Britt BB, Whitlock JA, Wilson JA. 2010.** First complete sauropod dinosaur skull from the Cretaceous of the Americas and the evolution of sauropod dentition. *Naturwissenschaften* **97:**379–391.

**Cohen KM, Finney SC, Gibbard PL, Fan J-X. 2013.** The ICS International Chronostratigraphic Chart. *Episodes* **36:**199–204.

**Comment G, Ayer J. 2010.** Définition macroscopique des unites lithostratigraphiques de l’Oxfordien moyen au Kimméridgien en Ajoie (Jura suisse): Nouvelles données acquises dans le cadre de la construction de l’autoroute A16. Report, Porrentruy, 24 pp.

**Contini D. 1989.** L'Oxfordien du Jura septentrional. Définition des formations. Evolution paléogéographique. *Annales Scientifiques de l’Université de Besançon. Géologie, 4e série* **9:**3–16.

**Cooper MR. 1981.** The prosauropod dinosaur *Massospondylus carinatus* Owen from Zimbabwe: its biology, mode of life and phylogenetic significance. *Occasional Papers of the National Museums and Monuments of Rhodesia, Series B, Natural Sciences* **6:**689–840.

**Courville P, Boullier A. 2014.** Les brachiopodes des “Calcaires et marnes à Spongiaires” (Oxfordien Moyen pp. – Oxfordien Supérieur pp.) du Sud-Est du Bassin parisien (France). *Fossiles. Hors-série* **V:**49–54.

**Curry Rogers K. 2005.** Titanosauria: a phylogenetic overview. In: Curry Rogers K, Wilson JA, eds. *The sauropods: evolution and paleobiology*. Berkeley; Los Angeles: University of California Press, 50–103.

**Curry Rogers K. 2009.** The postcranial osteology of *Rapetosaurus krausei* (Sauropoda: Titanosauria) from the Late Cretaceous of Madagascar. *Journal of Vertebrate Paleontology* **29:**1046–1086.

**Curry Rogers K, Forster CA. 2001.** The last of the dinosaur titans: a new sauropod from Madagascar. *Nature* **412:**530–534.

**Curry Rogers K, Forster C. 2004.** The skull of *Rapetosaurus krausei* (Sauropoda: Titanosauria) from the Late Cretaceous of Madagascar. *Journal of Vertebrate Paleontology* **24:**121–144.

**D’Emic MD. 2012.** The early evolution of titanosauriform sauropod dinosaurs. *Zoological Journal of the Linnean Society* **166:**624–671.

**D’Emic MD. 2013.** Revision of the sauropod dinosaurs of the Early Cretaceous Trinity Group, southern USA, with the description of a new genus. *Journal of Systematic Palaeontology* **11:**707–726.

**D’Emic MD, Wilson JA. 2011.** New remains attributable to the holotype of *Neuquensaurus australis* (Dinosauria: Sauropoda): implications for saltasaurine systematics. *Acta Palaeontologia Polonica* **56:**61–73.

**D’Emic MD, Foreman BZ, Jud NA. 2016.** Anatomy, systematics, paleoenvironment, growth, and age of the sauropod dinosaur *Sonorasaurus thompsoni* from the Cretaceous of Arizona, USA. *Journal of Paleontology* **90:**102–132.

**D’Emic MD, Wilson JA, Williamson TE. 2011.** A sauropod dinosaur pes from the latest Cretaceous of North America and the validity of *Alamosaurus sanjuanensis* (Sauropoda, Titanosauria). *Journal of Vertebrate Paleontology* **31:**1072–1079.

**D’Emic MD, Mannion PD, Upchurch P, Benson RBJ, Pang Q, Cheng Z. 2013.** Osteology of *Huabeisaurus allocotus* (Sauropoda: Titanosauriformes) from the Upper Cretaceous of China. *PLoS ONE* **8:**e69375.

**Dreyfuss M. 1934.** Sur une roche à ossements de Sauriens. *Bulletin de la Société d’histoire naturelle du Doubs* **44:**52–54.

**Enay R. 1966.** L’Oxfordien dans la moitié sud du Jura français: etude stratigraphique. *Nouvelles Archives du Muséum d’Histoire Naturelle de Lyon* **8:**1–624.

**Enay R. 1980.** Indices d’émersion et d’influences continentals dans l’Oxfordien supérieur-Kimméridgien inférieur en France. Interprétation paléogéographique et consequences paléobiogéographiques. *Bulletin de la Société Géologique de France, 7e série* **22:**581–590.

**Enay R, Contini D, Boullier A. 1988.** Le Séquanien-type de Franche-Comté (Oxfordien supérieur): datations et corrélations nouvelles, conséquences sur la paléogéographie et l'évolution du Jura et régions voisines. *Eclogae Geologicae Helvetiae* **81:**295–363.

**Gallina PA. 2011.** Notes on the axial skeleton of the titanosaur *Bonitasaura salgadoi* (Dinosauria-Sauropoda). *Anais da Academia Brasileira de Ciências* **83:**235–245.

**Gallina PA, Apesteguía S. 2005.** *Cathartesaura anaerobica* gen. et sp. nov., a new rebbachisaurid (Dinosauria, Sauropoda) from the Huincul Formation (Upper Cretaceous), Río Negro, Argentina. *Revista del Museo Argentino de Ciencias Naturales n.s.* **7:**153–166.

**Gallina PA, Apesteguía S. 2011.** Cranial anatomy and phylogenetic position of the titanosaurian sauropod *Bonitasaura salgadoi*. *Acta Palaeontologica Polonica* **56:**45–60.

**Garcia J-P, Philippe M, Gaumet F. 1998.** Fossil wood in Middle-Upper Jurassic marine sedimentary cycles of France: relations with climate, sea-level dynamics, and carbonate-platform environments. *Palaeogeography, Palaeoclimatology, Palaeoecology* **141:**199–214.

**Gauthier J. 1986.** Saurischian monophyly and the origin of birds. *Memoirs of the Californian Academy of Sciences* **8:**1–55.

**Głowniak E. 2006.** Correlation of the zonal schemes at the Middle–Upper Oxfordian boundary (Jurassic) in the Submediterranean Province: Poland and Switzerland. *Acta Geologica Polonica* **56:**33–50.

**Gomani EM. 2005.** Sauropod dinosaurs from the Early Cretaceous of Malawi. *Palaeontologia Electronica* **8:**1–37.

**González Riga BJ. 2003.** A new titanosaur (Dinosauria, Sauropoda) from the Upper Cretaceous of Mendoza Province, Argentina. *Ameghiniana* **40:**155–172.

**González Riga BJ. 2005.** Nuevos restos fósiles de *Mendozasaurus neguyelap* (Sauropoda, Titanosauridae) del Cretácico tardío de Patagonia. *Ameghiniana* **43:**535–548.

**González Riga BJ, Ortiz David L. 2014.** A New Titanosaur (Dinosauria, Sauropoda) from the Upper Cretaceous (Cerro Lisandro Formation) of Mendoza Province, Argentina. *Ameghiniana* **51:**3–25.

**González Riga BJ, Previtera E, Pirrone CA. 2009.** *Malarguesaurus florenciae* gen. et sp. nov., a new titanosauriform (Dinosauria, Sauropoda) from the Upper Cretaceous of Mendoza, Argentina. *Cretaceous Research* **30:**135–148.

**Gygi RA. 2000.** Integrated stratigraphy of the Oxfordian and Kimmeridgian (Late Jurassic) in northern Switzerland and adjacent southern Germany. Mémoires de l'Académie Suisse des Sciences Naturelles 104. Birkhäuser Verlag, Basel, 151 pp.

**Gygi RA. 2001.** Perisphinctacean ammonites of the type Transversarium Zone (Middle Oxfordian, Late Jurassic) in northern Switzerland. Mémoires Suisses de paléontologie 122. Kommission der Schweizerischen Paläontologischen Abhandlungen, Basel, 169 pp.

**Gygi RA. 2012.** Quantitative geology of Late Jurassic epicontinental sediments in the Jura Mountains of Switzerland. Springer Basel, Basel, 216 pp.

**Gygi RA, Persoz F. 1986.** Mineralostratigraphy, litho- and biostratigraphy combined in correlation of the Oxfordian (Late Jurassic) formations of the Swiss Jura range. *Eclogae Geologicae Helvetiae* **79:**385–454.

**Gygi RA, Coe AL, Vail PR. 1998.** Sequence stratigraphy of the Oxfordian and Kimmeridgian stages (Late Jurassic) in northern Switzerland. *SEPM Special Publication* **60:**527–544.

**Hardenbol J, Thierry J, Farley MB, Jacquin T, de Graciansky P-C, Vail PR. 1998.** Mesozoic and Cenozoic sequence chronostratigraphic framework of European basins. *SEPM Special Publication* **60:**3–13.

**Harris JD. 2006.** The significance of *Suuwassea emilieae* (Dinosauria: Sauropoda) for flagellicaudatan intrarelationships and evolution. *Journal of Systematic Palaeontology* **4:**185–198.

**Harris JD. 2007.** The appendicular skeleton of *Suuwassea emilieae* (Sauropoda: Flagellicaudata) from the Upper Jurassic Morrison Formation of Montana (USA). *Geobios* **40:**501–522.

**Jacobs LL, Winkler DA, Downs WR, Gomani EM. 1993.** New material of an Early Cretaceous titanosaurid sauropod dinosaur from Malawi. *Palaeontology* **36:**523–534.

**Janensch W. 1950.** Die wirbelsäule von *Brachiosaurus brancai*. *Palaeontographica (Supplement VII)* **3:**27–93.

**Kellner AWA, Campos DA, Trotta MNF. 2005.** Description of a titanosaurid caudal series from the Bauru Group, Late Cretaceous of Brazil. *Arquivos do Museu Nacional, Rio de Janeiro* **63:**529–564.

**Ksepka DT, Norell MA. 2010.** The illusory evidence for Asian Brachiosauridae: new material of *Erketu ellisoni* and a phylogenetic reappraisal of basal Titanosauriformes. *American Museum Novitates* **3700:**1–27.

**Lathuilière B, Gaillard C, Habrant N, Bodeur Y, Boullier A, Enay R, Hanzo M, Marchand D, Thierry J, Werner W. 2005.** Coral zonation of an Oxfordian reef tract in the northern French Jura. *Facies* **50:**545–559.

**Lü J, Xu L, Zhang X, Hu W, Wu Y, Jia S, Ji Q. 2007.** A new gigantic sauropod dinosaur with the deepest known body cavity from the Cretaceous of Asia. *Acta Geologica Sinica* **81:**167–176.

**Madsen J, McIntosh JS, Berman DS. 1995.** Skull and atlas–axis complex of the Upper Jurassic sauropod *Camarasaurus* Cope (Reptilia: Saurischia). *Bulletin of the Carnegie Museum of Natural History* **31:**1–115.

**Mannion PD. 2009.** A rebbachisaurid sauropod from the lower Cretaceous of the Isle of Wight, England. *Cretaceous Research* **30:**521–526.

**Mannion PD. 2011.** A reassessment of *Mongolosaurus haplodon* Gilmore, 1933, a titanosaurian sauropod dinosaur from the Early Cretaceous of Inner Mongolia, People’s Republic of China. *Journal of Systematic Palaeontology* **9:**355–378.

**Mannion PD, Calvo JO. 2011.** Anatomy of the basal titanosaur (Dinosauria, Sauropoda) *Andesaurus delgadoi* from the mid-Cretaceous (Albian-early Cenomanian) Río Limay Formation, Neuquén Province, Argentina: implications for titanosaur systematics. *Zoological Journal of the Linnean Society* **163:**155–181.

**Mannion PD, Upchurch P, Mateus O, Barnes R, Jones MEH. 2012.** New information on the anatomy and systematic position of *Dinheirosaurus lourinhanensis* (Sauropoda: Diplodocoidea) from the Late Jurassic of Portugal, with a review of European diplodocoids. *Journal of Systematic Palaeontology* **10:**521–551.

**Mannion PD, Upchurch P, Barnes RN, Mateus O. 2013.** Osteology of the Late Jurassic Portuguese sauropod dinosaur *Lusotitan atalaiensis* (Macronaria) and the evolutionary history of basal titanosauriforms. *Zoological Journal of the Linnean Society* **168:**98–206.

**Marpmann JS, Carballido JL, Sander PM, Knötschke N. 2015.** Cranial anatomy of the Late Jurassic dwarf sauropod *Europasaurus holgeri* (Dinosauria, Camarasauromorpha): ontogenetic changes and size dimorphism. *Journal of Systematic Palaeontology* **13:**221–263.

**Martínez RD, Giménez O, Rodríguez J, Luna M, Lamanna MC. 2004.** An articulated specimen of the basal titanosaurian (Dinosauria: Sauropoda) *Epachthosaurus sciuttoi* from the early Late Cretaceous Bajo Barreal Formation of Chubut province, Argentina. *Journal of Vertebrate Paleontology* **24**:107–120.

**Mateus O, Mannion PD, Upchurch P. 2014.** *Zby atlanticus*, a new turiasaurian sauropod (Dinosauria, Eusauropoda) from the Late Jurassic of Portugal. *Journal of Vertebrate Paleontology* **34:**618–634.

**McIntosh JS. 1990.** Sauropoda. In: Weishampel DB, Dodson P, Ósmolska H, eds. *The Dinosauria. First edn.* Berkeley, CA: University California Press, 345–401.

**Naish D, Martill DM. 2001.** Saurischian dinosaurs 1: Sauropods. In: Martill DM, Naish D, eds. *Dinosaurs of the Isle of Wight*. London: Palaeontological Association, 185–241.

**Otero A. 2010.** The appendicular skeleton of *Neuquensaurus*, a Late Cretaceous saltasaurine sauropod from Patagonia, Argentina. *Acta Palaeontologica Polonica* **55:**399–426.

**Paulina Carabajal A. 2012.** Neuroanatomy of titanosaurid dinosaurs from the Upper Cretaceous of Patagonia, with comments on endocranial variability within Sauropoda. *The Anatomical Record* **295:**2141–2156.

**Pernin C. 1978.** Etude géologique des abords du massif de la Serre. Thèse de 3e cycle, Université de Franche-Comté, Besançon, 162 pp.

**Poropat SF, Upchurch P, Mannion PD, Hocknull SA, Kear BP, Sloan T, Sinapius GHK, Elliott DA. 2015a.** Revision of the sauropod dinosaur *Diamantinasaurus matildae* Hocknull *et al*. 2009 from the middle Cretaceous of Australia: implications for Gondwanan titanosauriform dispersal. *Gondwana Research* **27:**995–1033.

**Poropat SF, Mannion PD, Upchurch P, Hocknull SA, Kear BP, Elliott DA. 2015b.** Reassessment of the non-titanosaurian somphospondylan *Wintonotitan wattsi* (Dinosauria: Sauropoda: Titanosauriformes) from the mid-Cretaceous Winton Formation, Queensland, Australia. *Papers in Palaeontology* **1:**59–106.

**Poropat SF, Mannion PD, Upchurch P, Hocknull SA, Kear BP, Kundrát M, Tischler TT, Sloan T, Sinapius GHK, Elliott JA, Elliott DA. 2016.** New Australian sauropods shed light on Cretaceous dinosaur palaeobiogeography. *Scientific Reports* **6:**34467.

**Powell JE. 1986.** Revision de los Titanosauridos de America del Sur. Unpublished Ph.D. dissertation, Universidad Nacional de Tucumán, Tucumán, Argentina.

**Powell JE. 1992.** Osteologia de *Saltasaurus loricatus* (Sauropoda-Titanosauridae) del Cretacico Superior del Noroeste argentino. In: Sanz JL, Buscalioni AD, eds. *Los dinosaurios y su entorno biotico*. Cuenca: Instituto Juan de Valdes, Serie Actas Academicas, 165–230.

**Powell JE. 2003.** Revision of South American titanosaurid dinosaurs: palaeobiological, palaeobiogeographical and phylogenetic aspects. *Records of the Queen Victoria Museum* **111:**1–173.

**Quereilhac P. 2013.** Étude de l'espèce Ochetoceras (Ochetoceras) canaliculatum (de BUCH, 1831) (Oxfordien moyen, Zone à Transversarium). *Carnets de Géologie* **13:**209–235.

**Rauhut OWM. 2006.** A brachiosaurid sauropod from the Late Jurassic Cañadón Calcàreo Formation of Chubut, Argentina. *Fossil Record* **9:**226–237.

**Rauhut OWM, Remes K, Fechner R, Cladera G, Puerta P. 2005.** Discovery of a short-necked sauropod dinosaur from the Late Jurassic period of Patagonia. *Nature* **435:**670–672.

**Remes K. 2009.** Taxonomy of Late Jurassic diplodocid sauropods from Tendaguru (Tanzania). *Fossil Record* **12:**23–46.

**Remes K, Ortega F, Fierro I, Joger U, Kosma R, Ferrer JMM, PALDES, SNHM, Ide OA, Maga A. 2009.** A new basal sauropod dinosaur from the Middle Jurassic of Niger and the early evolution of Sauropoda. *PLoS ONE* **4:**e6924.

**Rose PJ. 2007.** A new titanosauriform sauropod (Dinosauria: Saurischia) from the Early Cretaceous of central Texas and its phylogenetic relationships. *Palaeontologica Electronica* **10:**1–65.

**Royo-Torres R. 2009.** El saurópodo de Peñarroya de Tastavins. *Instituto de Estudios Turolenses-Fundación Conjunto Paleontológico de Teruel-Dinópolis, Monografías Turolenses* **6:**1–548.

**Royo-Torres R, Cobos A, Alcalá L. 2006.** A giant European dinosaur and a new sauropod clade. *Science* **314:**1925–1927.

**Royo-Torres R, Alcalá L, Cobos A. 2012.** A new specimen of the Cretaceous sauropod *Tastavinsaurus sanzi* from El Castellar (Teruel, Spain), and a phylogenetic analysis of the Laurasiformes. *Cretaceous Research* **34:**61–83.

**Royo-Torres R, Upchurch P, Mannion PD, Mas R, Cobos A, Gascó F, Alcalá L, Sanz JL. 2014.** The anatomy, phylogenetic relationships and stratigraphic position of the Tithonian–Berriasian Spanish sauropod dinosaur *Aragosaurus ischiaticus*. *Zoological Journal of the Linnean Society* **171:**623–655.

**Russell DA, Zheng Z. 1993.** A large mamenchisaurid from the Junggar Basin, Xinjiang, People’s Republic of China. *Canadian Journal of Earth Sciences* **30:**2082–2095.

**Salgado L, Calvo JO. 1997.** Evolution of titanosaurid sauropods. II: The cranial evidence. *Ameghiniana* **34:**33–47.

**Salgado L, Carvalho IS. 2008.** *Uberabatitan ribeiroi*, a new titanosaur from the Marília Formation (Bauru Group, Upper Cretaceous), Minas Gerais, Brazil. *Palaeontology* **51:**881–901.

**Salgado L, Powell JE. 2010.** Reassessment of the vertebral laminae in some South American titanosaurian sauropods. *Journal of Vertebrate Paleontology* **30:**1760–1772.

**Salgado L, Apesteguía S, Heredia SE. 2005.** A new specimen of *Neuquensaurus australis*, a Late Cretaceous saltasaurine titanosaur from north Patagonia. *Journal of Vertebrate Paleontology* **25**:623–634.

**Salgado L, Coria RA, Calvo JO. 1997.** Evolution of titanosaurid sauropods. I: phylogenetic analysis based on the postcranial evidence. *Ameghiniana* **34:**3–32.

**Sander PM, Mateus O, Laven T, Knötschke N. 2006.** Bone histology indicates insular dwarfism in a new Late Jurassic sauropod dinosaur. *Nature* **441:**739–741.

**Santucci RM, Arruda-Campos AC. 2011.** A new sauropod (Macronaria, Titanosauria) from the Adamantina Formation, Bauru Group, Upper Cretaceous of Brazil and the phylogenetic relationships of Aeolosaurini. *Zootaxa* **3085:**1–33.

**Sanz JL, Powell JE, Le Loueff J, Martinez R, Pereda Suberbiola X. 1999.** Sauropod remains from the Upper Cretaceous of Laño (northcentral Spain). Titanosaur phylogenetic relationships. *Estudios del Museo de Ciencias Naturales de Alava* **14:**235–255.

**Sekiya T. 2011.** Re-examination of *Chuanjiesaurus anaensis* (Dinosauria: Sauropoda) from the Middle Jurassic Chuanjie Formation, Lufeng County, Yunnan Province, southwest China. *Memoir of the Fukui Prefectural Dinosaur Museum* **10:**1–54.

**Sereno PC, Forster CA, Rogers RR, Monetta AM. 1993.** Primitive dinosaur skeleton from Argentina and the early evolution of Dinosauria. *Nature* **361:**64–66.

**Sereno PC, Wilson JA, Witmer LM, Whitlock JA, Maga A, Ide O, Rowe TA. 2007.** Structural extremes in a Cretaceous dinosaur. *PLoS ONE* **2:**e1230.

**Smith JB, Lamanna MC, Lacovara KJ, Dodson P, Smith JR, Poole JC, Giegengack R, Attia Y. 2001.** A giant sauropod dinosaur from an Upper Cretaceous mangrove deposit in Egypt. *Science* **292:**1704–1706.

**Strasser A. 2007.** Astronomical time scale for the Middle Oxfordian to Late Kimmeridgian in the Swiss and French Jura Mountains. *Swiss Journal of Geosciences* **100:**407–429.

**Suteethorn S, Le Loeuff J, Buffetaut E, Suteethorn V. 2010.** Description of topotypes of *Phuwiangosaurus sirindhornae*, a sauropod from the Sao Khua Formation (Early Cretaceous) of Thailand, and their phylogenetic implications. *Neues Jahrbuch für Geologie und Paläontologie – Abhandlungen* **256:**109–121.

**Taylor MP. 2009.** A re-evaluation of *Brachiosaurus altithorax* Riggs 1903 (Dinosauria, Sauropoda) and its generic separation from *Giraffatitan brancai* (Janensch 1914). *Journal of Vertebrate Paleontology* **29:**787–806.

**Tidwell V, Carpenter K, Meyer S. 2001.** New titanosauriform (Sauropoda) from the Poison Strip Member of the Cedar Mountain Formation (Lower Cretaceous), Utah. In: Tanke DH, Carpenter K, eds. *Mesozoic vertebrate life*. Bloomington, Indianapolis: Indiana University Press, 139–165.

**Tschopp E, Mateus O. 2013.** The skull and neck of a new flagellicaudatan sauropod from the Morrison Formation and its implication for the evolution and ontogeny of diplodocid dinosaurs. *Journal of Systematic Palaeontology* **11:**853–888.

**Tschopp E, Mateus O, Benson RBJ 2015.** A specimen-level phylogenetic analysis and taxonomic revision of Diplodocidae (Dinosauria, Sauropoda). *PeerJ* **3:**e857.

**Upchurch P. 1995.** The evolutionary history of sauropod dinosaurs. *Philosophical Transactions of the Royal Society of London, Series B* **349:**365–390.

**Upchurch P. 1998.** The phylogenetic relationships of sauropod dinosaurs. *Zoological Journal of the Linnean Society* **124:**43–103.

**Upchurch P. 1999.** The phylogenetic relationships of the Nemegtosauridae (Saurischia, Sauropoda). *Journal of Vertebrate Paleontology* **19:**106–125.

**Upchurch P, Martin J. 2002.** The Rutland *Cetiosaurus*: the anatomy and relationships of a Middle Jurassic British sauropod dinosaur. *Palaeontology* **45:**1049–1074.

**Upchurch P, Martin J. 2003.** The anatomy and taxonomy of *Cetiosaurus* (Saurischia: Sauropoda) from the Middle Jurassic of England. *Journal of Vertebrate Paleontology* **23:**208–231.

**Upchurch P, Barrett PM, Dodson P. 2004.** Sauropoda. In: Weishampel DB, Dodson P, Osmólska H, eds. *The Dinosauria, 2nd edn.* Berkeley: University of California Press, 259–324.

**Upchurch P, Barrett PM, Galton PM. 2007.** The phylogenetic relationships of basal sauropodomorphs: implications for the origin of sauropods. *Special Papers in Palaeontology* **77:**57–90.

**Upchurch P, Mannion PD, Taylor MP. 2015.** The Anatomy and Phylogenetic Relationships of “*Pelorosaurus*“ *becklesii* (Neosauropoda, Macronaria) from the Early Cretaceous of England. *PLoS ONE* **10:**e0125819.

**Wedel MJ, Cifelli RL, Sanders RK. 2000.** *Sauroposeidon proteles*, a new sauropod from the Early Cretaceous of Oklahoma. *Journal of Vertebrate Paleontology* **20:**109–114.

**Whitlock JA. 2011.** A phylogenetic analysis of Diplodocoidea (Saurischia: Sauropoda). *Zoological Journal of the Linnean Society* **161:**872–915.

**Whitlock JA, D’Emic MD, Wilson JA. 2011.** Cretaceous diplodocids in Asia? Re-evaluating the phylogenetic affinities of a fragmentary specimen. *Palaeontology* **54:**351–364.

**Whitlock JA, Wilson JA, Lamanna MC. 2010.** Description of a nearly complete juvenile skull of *Diplodocus* (Sauropoda: Diplodocoidea) from the Late Jurassic of North America. *Journal of Vertebrate Paleontology* **30:**442–457.

**Wilson JA. 1999.** A nomenclature for vertebral laminae in sauropods and other saurischian dinosaurs. *Journal of Vertebrate Paleontology* **19:**639–653.

**Wilson JA. 2002.** Sauropod dinosaur phylogeny: critique and cladistic analysis. *Zoological Journal of the Linnean Society* **136:**217–276.

**Wilson JA. 2005.** Redescription of the Mongolian sauropod *Nemegtosaurus mongoliensis* Nowinski (Dinosauria: Saurischia) and comments on Late Cretaceous sauropod diversity. *Journal of Systematic Palaeontology* **3:**283–318.

**Wilson JA, Carrano MT. 1999.** Titanosaurs and the origin of ‘wide-gauge’ trackways: a biomechanical and systematic perspective on sauropod locomotion. *Paleobiology* **25:**252–267.

**Wilson JA, Sereno PC. 1998.** Early evolution and higher-level phylogeny of sauropod dinosaurs. *Society of Vertebrate Paleontology Memoir* **5:**1–68.

**Wilson JA, Upchurch P. 2009.** Redescription and reassessment of the phylogenetic affinities of *Euhelopus zdanskyi* (Dinosauria: Sauropoda) from the Early Cretaceous of China. *Journal of Systematic Palaeontology* **7:**199–239.

**Wilson JA, D’Emic MD, Ikejiri, T, Moacdieh EM, Whitlock JA. 2011.** A nomenclature for vertebral fossae in sauropods and other saurischian dinosaurs. *PLoS ONE* **6:**e17114.

**Yates AM. 2007.** The first complete skull of the Triassic dinosaur *Melanorosaurus* Haughton (Sauropodomorpha: Anchisauria). *Special Papers in Palaeontology* **77:**9–55.

**Yates AM, Kitching J. 2003.** The earliest known sauropod dinosaur and the first steps towards sauropod locomotion. *Proceedings of the Royal Society of London, Series B* **270:**1753–1758.

**Zaher H, Pol D, Carvalho AB, Nascimento PM, Riccomini C, Larson P, Juarez-Valieri R, Pires-Domingues R, Silva NJ, Campos DA. 2011.** A complete skull of an Early Cretaceous sauropod and the evolution of advanced titanosaurians. *PLoS ONE* **6:**e16663.

**Figure S1. Palaeogeography of the carbonated Jura platform during the upper Oxfordian–lower Kimmeridgian.** Map modified after Cariou (2013). Abbreviated locations for: (1) truncations: O: Ornans (Enay, Contini & Boullier, 1988), BP: Bonnevaux-le-Prieuré (Lathuilière *et al*., 2005), Damparis (this study); and (2) fossil woods: BC: Besançon (Citadelle) (Bulle *et al*., 1968): characean algae horizons; BG: Besançon (Brégille) (Contini, 1972): basal black marls with *Brachyphyllum moreauanum*, imprints of *Zamites* and *Zonarites gracillimus*; CS: Charbonnière-les-Sapins (Merle, 1905): lignite horizon with characean algea separating the Rauracian from the Sequanian, with Zamites and characean pinnules; D: Dambelin (Enay, Contini & Boullier, 1988): grey-black marls; Gi: Gilley (Oertli & Ziegler, 1958): gyrogonites of characean algae and shells of limnic ostracods from freshwater ponds; Gr: Grattery (Contini, 1972): ~10 m above the base: vegetation imprints: fronds and pinnules of *Zamites feneonis* and *Zamites formosus*, pieces of leaf stems of *Brachyphyllum moreauanum*; H: Hyémondans (Enay, Contini & Boullier, 1988): basal black marls with wood debris; RV: Roche-sur-Vannon (Glangeaud, 1944): *Zamites feneonis*, *Brachyphyllum moreauanum*, *Cycadospermum* (fruits), young pinecones of evergreens, algae.

**Table S1. Correlation scheme of the lithological units of Damparis with both synthetic geological successions of Franche-Comté region and of north-western Switzerland.**

**Table S2. Biostratigraphical distinction of the geological formations from the late Middle and Upper Oxfordian in Franche-Comté (France).** Taxon names and stratigraphical ranges have been updated from original publications and different sources respectively. Only two taxa (highlighted in yellow) are still affected by taxonomic vs. stratigraphic inconsistencies.
